# Pharmacological activation of ATF6 remodels the proteostasis network to rescue pathogenic GABA_A_ receptors

**DOI:** 10.1186/s13578-022-00783-w

**Published:** 2022-04-27

**Authors:** Meng Wang, Edmund Cotter, Ya-Juan Wang, Xu Fu, Angela L. Whittsette, Joseph W. Lynch, R. Luke Wiseman, Jeffery W. Kelly, Angelo Keramidas, Ting-Wei Mu

**Affiliations:** 1 Department of Physiology and Biophysics, Case Western Reserve University School of Medicine, 10900 Euclid Ave, Cleveland, OH 44106 USA; 2 Queensland Brain Institute, the University of Queensland, Brisbane, QLD 4072 Australia; 3 Department of Molecular Medicine, The Scripps Research Institute, 10550 North Torrey Pines Road, La Jolla, CA 92037 USA

## Abstract

**Background:**

Genetic variants in the subunits of the gamma-aminobutyric acid type A (GABA_A_) receptors are implicated in the onset of multiple pathologic conditions including genetic epilepsy. Previous work showed that pathogenic GABA_A_ subunits promote misfolding and inefficient assembly of the GABA_A_ receptors, limiting receptor expression and activity at the plasma membrane. However, GABA_A_ receptors containing variant subunits can retain activity, indicating that enhancing the folding, assembly, and trafficking of these variant receptors offers a potential opportunity to mitigate pathology associated with genetic epilepsy.

**Results:**

Here, we demonstrate that pharmacologically enhancing endoplasmic reticulum (ER) proteostasis using small molecule activators of the ATF6 (Activating Transcription Factor 6) signaling arm of the unfolded protein response (UPR) increases the assembly, trafficking, and surface expression of variant GABA_A_ receptors. These improvements are attributed to ATF6-dependent remodeling of the ER proteostasis environment, which increases protein levels of pro-folding ER proteostasis factors including the ER chaperone BiP (Immunoglobulin Binding Protein) and trafficking receptors, such as LMAN1 (Lectin Mannose-Binding 1) and enhances their interactions with GABA_A_ receptors. Importantly, we further show that pharmacologic ATF6 activators increase the activity of GABA_A_ receptors at the cell surface, revealing the potential for this strategy to restore receptor activity to levels that could mitigate disease pathogenesis.

**Conclusions:**

These results indicate that pharmacologic ATF6 activators offer an opportunity to restore GABA_A_ receptor activity in diseases including genetic epilepsy and point to the potential for similar pharmacologic enhancement of ER proteostasis to improve trafficking of other disease-associated variant ion channels implicated in etiologically-diverse diseases.

**Supplementary Information:**

The online version contains supplementary material available at 10.1186/s13578-022-00783-w.

## Introduction

Gamma-aminobutyric acid type A (GABA_A_) receptors are located at synaptic sites and mediate the majority of fast inhibitory neurotransmission in the mammalian brain. GABA_A_ receptor activation by the neurotransmitter, GABA, leads to membrane hyperpolarization of the post-synaptic (target) neuron and suppression of neuronal activity [1, 2]. Dysfunction of GABA_A_ receptors results in a variety of neurological and neurodevelopmental disorders, including anxiety disorders, autism, and epilepsy [3]. Accumulating evidence indicates that a key pathological mechanism for many GABA_A_ receptor variants is reduced or ablated surface expression of receptors, particularly at synaptic sites [4–7]. For example, a missense mutation D219N in the α1 subunit, which was identified in a cohort of patients with idiopathic generalized epilepsy, impaired the membrane delivery of the mature GABA_A_ receptor [8]. Two missense mutations R177G and R82Q in the γ2 subunit, which were reported in patients with febrile seizures and childhood absence epilepsy, have been demonstrated to decrease GABA_A_ receptor surface expression [9–12]. Additional file 1: Figure S1A represents the positions of these mutations in the cryo-electron microscopy (cryo-EM) structure of pentameric GABA_A_ receptors [13]. These results demonstrate that reduced surface expression of mutant GABA_A_ receptors is a key determinant in the pathogenesis of these neurological diseases. Therefore, restoring variant-containing GABA_A_ receptor expression, and thus function, is a potential therapeutic strategy.

An attractive strategy to increase folding, assembly, and trafficking of variant-containing GABA_A_ receptors is to enhance proteostasis capacity in the endoplasmic reticulum (ER). Previous results have shown that suberoylanilide hydroxamic acid (SAHA), dinoprost, and dihydroergocristine promoted the folding and trafficking of the α1(A322D) variant by enhancing the interaction between the variant and ER chaperones, BiP and calnexin [14, 15]. In addition, L-type channel blockers, such as verapamil, were demonstrated to enhance the functional surface expression of the α1(D219N) variant by promoting the activity of calcium-dependent ER chaperone calnexin [16]. This suggests that pharmacologic enhancement of ER proteostasis offers an opportunity to improve the trafficking and membrane activity of variant-containing GABA_A_ receptors [17].

One potential method to enhance ER proteostasis is through pharmacological activation of the unfolded protein response (UPR) [18]. The UPR comprises three signaling arms activated downstream of the ER membrane proteins IRE1 (Inositol-Requiring Enzyme 1), ATF6 (Activating Transcription Factor 6), and PERK (Protein Kinase R-like ER Kinase) [19, 20]. In response to ER stress, these pathways are activated, resulting in both translational and transcriptional signaling that functions to regulate diverse aspects of cellular physiology including ER proteostasis. Notably, the ATF6 arm of the UPR regulates the expression of numerous ER proteostasis factors that include BiP, calnexin, and LMAN1, which are critical for the folding, assembly and trafficking of GABA_A_ subunits [14, 16, 21, 22]. This suggests that pharmacologic enhancement of ATF6 activation offers a therapeutic strategy for improving the trafficking of variant-containing GABA_A_ receptors and their function at the cell surface.

Recently, ER proteostasis regulators including AA147 and AA263 were demonstrated to enhance ER proteostasis through preferential activation of the ATF6 arm of the UPR [23]. ATF6 is activated in response to ER stress through a mechanism involving reduction and monomerization of ATF6 oligomers, increased trafficking to the Golgi, and subsequent proteolytic processing by Site 1 and Site 2 proteases (S1P and S2P, respectively) [24]. This releases the active N-terminal ATF6 transcription factor domain that localizes to the nucleus and promotes induction of ATF6 responsive genes. AA147 and AA263 activate ATF6 through a mechanism involving compound metabolic activation and covalent modification of ER-localized protein disulfide isomerases (PDIs), increasing the population of reduced monomeric ATF6 available for trafficking to the Golgi and proteolytic activation [25]. These compounds have been widely used to investigate the potential for pharmacologic ATF6 activation as a method of mitigating pathology associated with etiologically diverse diseases, such as myocardial infarction, cardiac arrest, and achromatopsia [26–28].

Here, we employed AA147 and AA263 to investigate how pharmacologic activation of ATF6 enhances the folding, assembly, and trafficking of variant-containing GABA_A_ receptors. We reported that AA147 and AA263 increase the total and surface protein levels and restore synaptic function of variant receptors by reprogramming the ER proteostasis network to promote the folding and forward trafficking of variant receptors.

## Results

### ATF6 activators increase the total and surface protein levels of a trafficking-deficient α1(D219N) subunit of GABA_A_ receptors

**Fig. 1:**
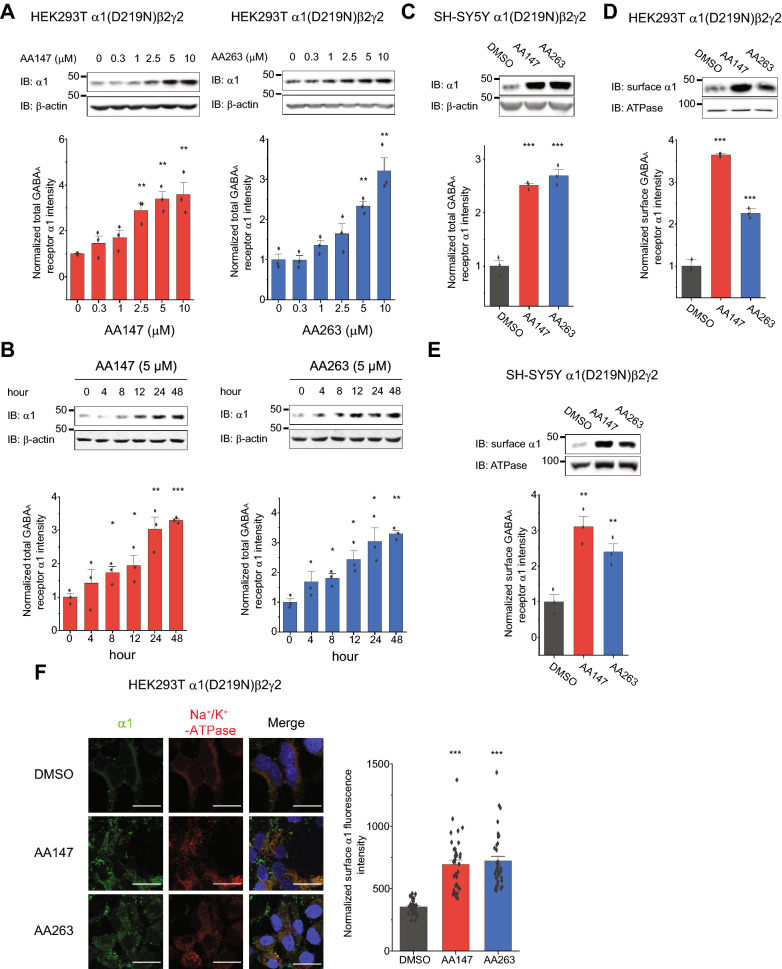
Effect of AA147 and AA263 on the total and surface protein levels of trafficking-deficient mutant GABA_A_ receptors. **A** Dose–response analysis of AA147 and AA263 treatment (24 h) on the total protein levels of α1(D219N) subunits in HEK293T cells expressing α1(D219N)β2γ2 GABA_A_ receptors. **B** Time-course analysis of AA147 (5 µM) and AA263 (5 µM) treatment on the total protein levels of α1(D219N) subunits in HEK293T cells expressing α1(D219N)β2γ2 GABA_A_ receptors. **C** Effect of AA147 (2.5 µM, 24 h) and AA263 (2.5 µM, 24 h) on the total protein level of α1(D219N) subunits in neuronal SH-SY5Y cells stably expressing α1(D219N)β2γ2 GABA_A_ receptors. β-actin serves as total protein loading control. **D** Effect of AA147 (5 µM, 24 h) and AA263 (5 µM, 24 h) on the surface protein expression of the α1(D219N) subunits in HEK293T cells stably expressing α1(D219N)β2γ2 GABA_A_ receptors according to surface biotinylation analysis. **E** Effect of AA147 (2.5 µM, 24 h) and AA263 (2.5 µM, 24 h) on the surface protein expression of the α1(D219N) subunits in neuronal SH-SY5Y cells stably expressing α1(D219N)β2γ2 GABA_A_ receptors according to surface biotinylation analysis. Na^+^/K^+^-ATPase serves as membrane protein loading control. Quantification of the band intensities is shown on the bottom panels (n = 3). **F** HEK293T cells stably expressing α1(D219N)β2γ2 receptors were treated with DMSO vehicle control, AA147 (5 µM, 24 h) or AA263 (5 µM, 24 h). Surface α1 staining was in green (column 1), and plasma membrane marker Na^+^/K^+^-ATPase staining in red (column 2). Merge of these two signals and nucleus staining by DAPI in blue was shown in column 3. Scale bar = 20 μm. Quantification of the fluorescence intensity of the surface subunits from 30–40 cells per condition is shown on the right. IB: immunoblotting. Each data point is reported as mean ± SEM. One-way ANOVA followed by post-hoc Tukey test was used for statistical analysis. *, *p* < 0.05; **, *p* < 0.01; ***, *p* < 0.001

Recent studies have demonstrated that the stress-independent activators of the ATF6 pathway, AA147 and AA263, reduce secretion and toxic extracellular aggregation of destabilized, aggregation-prone proteins [23]. However, whether these activators can restore the trafficking of misfolding-prone proteins is still unclear. To answer this question, we applied AA147 or AA263 to cells expressing one well-characterized pathogenic GABA_A_ receptor variant that is prone to misfolding and excessive degradation, namely the D219N variant in the α1 subunit [8, 16]. HEK293T cells stably expressing α1(D219N)β2γ2 GABA_A_ receptors were treated with a range of concentrations of AA147 or AA263 from 0.3 to 10 μM for 24 h. We then monitored steady-state protein levels of GABA_A_ receptors by immunoblotting. AA147 and AA263 significantly increased steady-state α1(D219N) protein levels at concentrations above 2.5 μM or 5 μM (Fig. 1A). Therefore, in the following experiments, we used 5 μM ATF6 activators to maximize the response without apparent cellular toxicity in HEK293T cells. Time-course experiments demonstrated that a single dose application of AA147 and AA263 significantly increased the total α1(D219N) protein levels after 8 h (Fig. 1B), consistent with the time scale for the activation of the ATF6 pathway [23] as well as for the folding and trafficking process of GABA_A_ receptors [14]. In addition, such an increase maximized at 24 h and maintained at least until 48 h post treatment (Fig. 1B). In addition, dose–response experiments in neuronal SH-SY5Y cells stably expressing α1(D219N) β2γ2 GABA_A_ receptors showed that the effect of AA147 and AA263 on increasing the total α1 protein levels maximized at 2.5 μM (Additional file 1: Figure S2A) without apparent cell toxicity (Additional file 1: Figure S2B). Therefore, we used 2.5 μM ATF6 activators in neuronal SH-SY5Y cells in the following experiments (Fig. 1C).

Furthermore, dose–response experiments showed that AA147 and AA263 had minimal effects on the total protein levels of wild-type α1 subunits in HEK293T cells (Additional file 1: Fig. S3A, B), consistent with previous results showing that these compounds do not globally influence secretion of stable, endogenous proteins [23]. Moreover, compared to the DMSO vehicle control, AA147 and AA263 did not change the apparent cell morphology in HEK293T cells expressing wild-type α1β2γ2, α1(D219N)β2γ2, or α1β2γ2(R177G) GABA_A_ receptors (Additional file 1: Figure S3C). In addition, quantitative reverse-transcription polymerase chain reaction (qRT-PCR) demonstrated that AA147 and AA263 did not substantially change the mRNA levels of α1(D219N) (Additional file 1: Figure S3D), indicating their post-transcriptional effect on α1(D219N) protein levels. Since we used polyclonal stable cells expressing GABA_A_ variants, caution needs to be taken about the heterogeneity of the cell populations, which could be differentially affected by AA147 and AA263.

Because GABA_A_ receptors need to be transported to synaptic sites at the cell membrane, we determined whether ATF6 activators affected the cell surface expression of variant receptors. Surface biotinylation assays clearly demonstrated that AA147 and AA263 increased the surface expression of α1(D219N) in HEK293T cells (Fig. 1D), as well as in neuronal SH-SY5Y cells (Fig. 1E). Furthermore, we performed indirect immunofluorescence microscopy experiments to determine how drug treatments affected the surface staining of variant subunits. The application of an anti-GABA_A_ receptor subunit antibody that recognizes the extracellular epitope without a prior membrane permeabilization step enabled us to specifically label cell surface proteins. Consistent with the surface biotinylation results, the confocal microscopy data demonstrated that AA147 and AA263 increased the surface staining of α1(D219N) subunits in HEK293T cells, which merged well with the staining of a plasma membrane marker Na^+^/K^+^-ATPase (Fig. 1F). The above data demonstrated that ATF6 activators enhanced steady-state protein levels and surface expression of a misfolding-prone mutant subunit of GABA_A_ receptors.

### ATF6 activators enhance folding and forward trafficking of defective receptors

Subsequently, we elucidated the mechanism underlying AA147 and AA263 mediated rescue of misfolding-prone α1(D219N) subunit. We first determined whether the effect of AA147 and AA263 on GABA_A_ receptor proteostasis depends on the activation of the ATF6 pathway using potent inhibitors of the ATF6 pathway, including Ceapin-A7, which retains ATF6α in the ER [29], and PF429242, which inhibits Site 1 protease (S1P) [30]. Co-treatment of HEK293T cells stably expressing α1(D219N)β2γ2 receptors with AA147 and Ceapin-A7 reduced the increase of the α1(D219N) steady-state protein levels afforded by AA147 treatment alone (Additional file 1: Figure S4A, cf. lane 4 to lane 3). Similar results were observed upon co-treatment of AA147 with PF429242 (Additional file 1: Figure S4B, cf. lane 4 to lane 3). Ceapin-A7 (Additional file 1: Figure S4C) and PF429242 (Additional file 1: Figure S4D) also attenuated the AA263-dependent increase of α1(D219N) protein levels. These results indicated that AA147- and AA263-dependent ATF6 activation contributes to the restored proteostasis of mutant GABA_A_ receptors. Since inhibiting ATF6 only partially blocked the effect of AA147 and AA263 on α1(D219N) protein levels, other cellular signaling pathways could also contribute. Therefore, since the IRE1 arm and the ATF6 arm of the UPR are known to regulate the ER protein folding environment [18, 19], we continued to determine whether inhibiting IRE1 influenced the effect of AA147 and AA263. Co-application of 4μ8c, a potent IRE1 inhibitor acting on the RNase domain [31], did not diminish the increase of α1(D219N) proteins afforded by AA147 or AA263 significantly (Additional file 1: Figure S4E), indicating that the IRE1 pathway is not critical in the activity of AA147 and AA263. Nonetheless, other cellular signaling pathways that are involved in the activity of AA147 and AA263 merit future investigation.

**Fig. 2:**
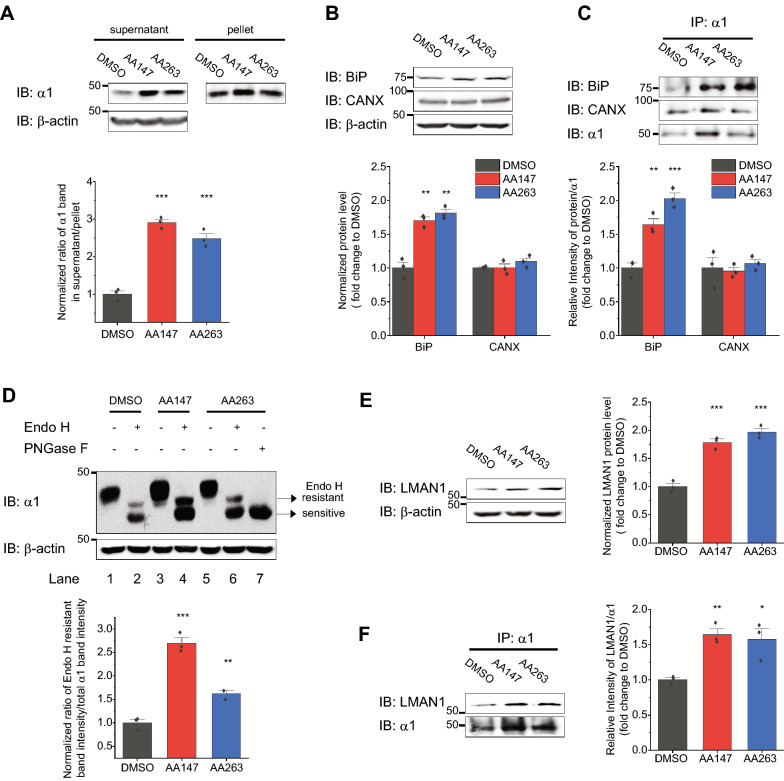
AA147 and AA263 enhance the folding and trafficking of the α1(D219N) subunit. **A** Effect of AA147 (5 µM, 24 h) and AA263 (5 µM, 24 h) on the folding of α1(D219N) subunits in HEK293T cells stably expressing α1(D219N)β2γ2 GABA_A_ receptors. Cells were lysed in Tris-buffered saline with 2 mM *n*-Dodecyl-β-D-maltoside (DDM) supplemented with Roche protease inhibitor cocktail. The detergent insoluble fractions were re-suspended with SDS sample loading buffer and then subjected to SDS-PAGE and western blot analysis. Quantification of the detergent soluble /insoluble fractions is shown on the bottom panels (n = 3). **B** Effect of AA147 (5 µM, 24 h) and AA263 (5 µM, 24 h) on steady-state protein levels of BiP and calnexin (CANX) in HEK293T cells stably expressing α1(D219N)β2γ2 GABA_A_ receptors. Quantifications of the normalized band intensities are shown on the bottom (n = 3). **C** Effect of AA147 (5 µM, 24 h) and AA263 (5 µM, 24 h) on the interactions between α1(D219N) subunits and BiP and calnexin in HEK293T cells expressing α1(D219N)β2γ2 receptors (*n* = 3). Apyrase (10 units / mL), which hydrolyzes ATP, was added to the co-immunoprecipitation buffer during the co-immunoprecipitation experiments to enhance the detection of the interactions between BiP and α1(D219N). Quantification of the ratio of the target proteins and α1(D219N) post immunoprecipitation (IP) is shown in the bottom panels. **D** AA147 (5 µM, 24 h) and AA263 (5 µM, 24 h) increases the Endo H-resistant post-ER glycoform of the α1(D219N) subunit in HEK293T cells stably expressing α1(D219N)β2γ2 GABA_A_ receptors. PNGase F, which cleaves all glycans from a glycoprotein, is included to indicate the unglycosylated α1 subunits. Quantification of the Endo H resistant/total α1 band intensities is shown on the bottom panels (n = 3). **E** Effect of AA147 (5 µM, 24 h) and AA263 (5 µM, 24 h) on steady-state protein levels of LMAN1 in HEK293T cells stably expressing α1(D219N)β2γ2 GABA_A_ receptors. Quantifications of the normalized band intensities are shown on the right (n = 3). **F** Effect of AA147 (5 µM, 24 h) and AA263 (5 µM, 24 h) on the interactions between α1(D219N) subunits and LMAN1 in HEK293T cells expressing α1(D219N)β2γ2 receptors (*n* = 3). Quantification of the ratio of the target proteins and α1(D219N) post immunoprecipitation is shown on the right. Each data point is reported as mean ± SEM. One-way ANOVA followed by post-hoc Tukey test was used for statistical analysis. *, *p* < 0.05; **, *p* < 0.01; ***, *p* < 0.001

Thereafter, we explored whether compound treatment enhanced folding and facilitated trafficking of α1(D219N). Since the detergent *n*-Dodecyl-β-d-maltoside (DDM) was effective in solubilizing GABA_A_ receptors [13], we used a DDM (2 mM) detergent solubility assay to quantify the relative folding extent of α1(D219N) in HEK293T cells by determining the ratio of its supernatant/pellet fraction. Clearly, AA147 and AA263 treatments increased this ratio for the α1(D219N) variant (Fig. 2A), indicating that ATF6 activators re-partitioned mutant proteins from an aggregation-prone state to a pro-folding state. Because the proteostasis network is critical in regulating the biogenesis of GABA_A_ receptors [32], we determined how pharmacological activation of the ATF6 pathway affected GABA_A_ receptor folding network. Previously, we demonstrated that BiP, a 70-kDa heat shock protein (Hsp70) family member chaperone in the ER, and calnexin, a lectin chaperone in the ER, both play an important role in facilitating the productive folding of GABA_A_ receptors [14, 16]. Therefore, we evaluated how AA147 and AA263 influenced these pro-folding chaperones in HEK293T cells stably expressing α1(D219N)β2γ2 receptors. AA147 and AA263 significantly increased BiP protein levels (Fig. 2B), consistent with their role as ATF6 activators, whereas they had no effect on calnexin protein levels (Fig. 2B). In addition, a co-immunoprecipitation assay revealed that AA147 and AA263 enhanced the interaction between BiP and α1(D219N), whereas they did not change the interaction between calnexin and α1(D219N) (Fig. 2C).

Moreover, we evaluated whether enhanced folding of α1(D219N) resulted in its productive anterograde trafficking. An endoglycosidase H (Endo H) enzyme digestion assay was used to quantify the trafficking efficiency of α1(D219N) from the ER to the Golgi. Endo H resistant α1(D219N) subunits indicated that they exit the ER and traffic at least to the Golgi, whereas Endo H sensitive α1(D219N) subunits are retained in the ER. The ratio of Endo H resistant / sensitive bands is a measure of the trafficking efficiency of α1(D219N) subunits. The Endo H enzyme digestion assay showed that AA147 and AA263 prominently increased this ratio (Fig. 2D, cf. lanes 4 and 6 to lane 2), indicating that these compounds promoted correctly folded, post-ER glycoforms of α1(D219N) subunits. Previously, we showed that ER-Golgi intermediate compartment 53 kDa protein (ERGIC53, otherwise known as LMAN1) plays an important role in transporting mature GABA_A_ receptors from the ER to the Golgi in the central nervous system [21]. Western blot analysis showed that AA147 and AA263 increased the total protein level of LMAN1 (Fig. 2E), indicating that they utilized LMAN to promote the ER-to-Golgi trafficking of α1(D219N)-containing receptors. In addition, a co-immunoprecipitation assay demonstrated that AA147 and AA263 increased the interaction between LMAN1 and α1(D219N) (Fig. 2F). Therefore, these compounds maintain the variant subunits in a folding-competent state by up-regulating BiP and enhance their forward trafficking by upregulating LMAN1.

### AA147 and AA263 reduce the degradation of defective receptors

**Fig. 3:**
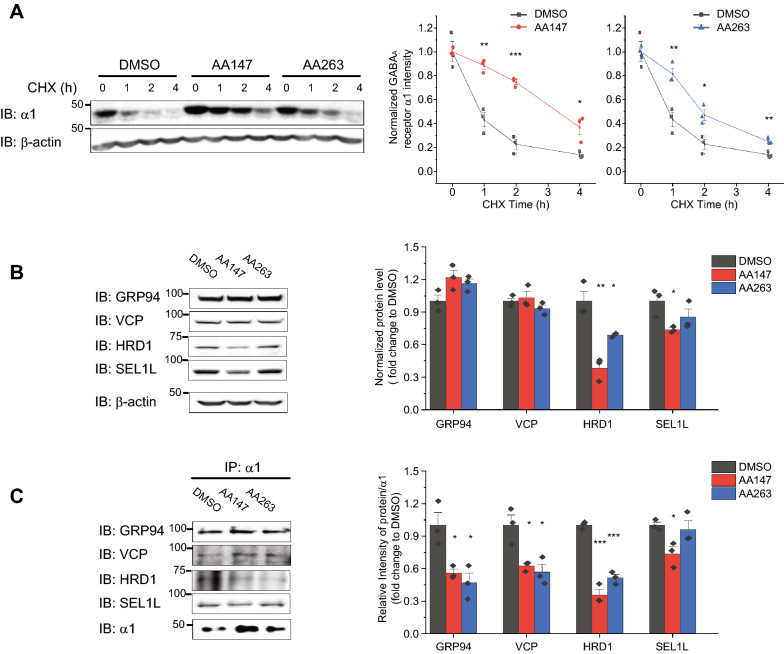
AA147 and AA263 reduce the degradation of the α1(D219N) subunit. **A** Effect of AA147 (5 µM, 24 h) and AA263 (5 µM, 24 h) on the degradation of the α1(D219N) subunit in HEK293T cells stably expressing α1(D219N)β2γ2 GABA_A_ receptors using cycloheximide (CHX)-chase analysis. **B** Effect of AA147 (5 µM, 24 h) and AA263 (5 µM, 24 h) on steady-state protein levels of ERAD factors, including GRP94, VCP, HRD1, and SEL1L in HEK293T cells stably expressing α1(D219N)β2γ2 GABA_A_ receptors. Quantification of the normalized band intensities is shown on the right (n = 3). **C** Effect of AA147 (5 µM, 24 h) and AA263 (5 µM, 24 h) on the interactions between α1(D219N) subunits and ERAD factors in HEK293T cells expressing α1(D219N)β2γ2 receptors. Quantification of the ratio of the target proteins and α1(D219N) post immunoprecipitation (IP) is on the right (n = 3). Each data point is reported as mean ± SEM. For statistical analysis, two-tailed Student’s t-test was used in (**A**), whereas one-way ANOVA followed by post-hoc Tukey test was used in **B** and **C**. *, *p* < 0.05; **, *p* < 0.01; ***, *p* < 0.001

Owing to the fact that the ATF6 pathway is closely connected with the ER-associated degradation (ERAD) pathway and the α1(D219N) subunits are rapidly degraded by ERAD [16], we determined how AA147 and AA263 regulated the degradation of variant GABA_A_ subunits. We used cycloheximide (CHX) to inhibit protein synthesis and monitored the protein stability of α1(D219N) as a function of time in cells treated with AA147 or AA263. This experiment showed that AA147 and AA263 attenuated the degradation of the α1(D219N) subunit in HEK293T cells (Fig. 3A).

Furthermore, we determined how AA147 and AA263 influenced ERAD factors to reduce α1(D219N) subunit degradation. Previously, we showed that GRP94, a 90-kDa heat shock protein (Hsp90) family chaperone in the ER lumen, directs misfolded α1 subunits to the ERAD pathway [33]. We also demonstrated that the mammalian central ubiquitin E3 ligase complex containing HMG-CoA reductase degradation protein 1 (HRD1) and Suppressor/Enhancer of Lin-12-like protein 1 (SEL1L) ubiquitinates α1 subunits, which are then extracted from the ER membrane to the cytosol for degradation by valosin-containing protein (VCP), an ATPase. Although AA147 and AA263 did not significantly change total protein levels of GRP94 and VCP (Fig. 3B), a co-immunoprecipitation assay showed that they reduced the interactions between α1(D219N) and GRP94/VCP (Fig. 3C). In addition, AA147 and AA263 significantly decreased total protein levels of HRD1 (Fig. 3B) as well as the interactions between α1(D219N) and HRD1 (Fig. 3C). Notably, AA147 reduced the total protein level of SEL1L (Fig. 3B) and the interaction between α1(D219N) and SEL1L (Fig. 3C), but AA263 could not, suggesting a subtle difference between the effects of AA147 and AA263 on the ERAD network. These results indicated that AA147 and AA263 inhibited the GRP94-HRD1/VCP mediated ERAD pathway of α1(D219N) to reduce the degradation of the misfolding-prone mutant subunit.

### AA147 and AA263 enhance the surface expression of a variety of trafficking-deficient variant GABA_A_ receptors

Because activating the ATF6 pathway pharmacologically provides a general strategy for handling misfolded proteins, we sought to determine whether AA147 and AA263 are effective in rescuing a multitude of trafficking-deficient variant GABA_A_ receptors. We tested two pathogenic variants in the γ2 subunits: R82Q [10] and R177G [11]. Dose–response experiments showed that AA147 (Additional file 1: Figure S5A) and AA263 (Additional file 1: Figure S5B) significantly increased γ2(R177G) total protein levels in HEK293T cells. Similar effects were observed for γ2(R82Q) total protein levels in HEK293T cells (Additional file 1: Figures S5C and D). In addition, we evaluated the effect of AA147 and AA263 in SH-SY5Y cells stably expressing α1β2γ2(R177G) or α1β2γ2(R82Q) GABA_A_ receptors. Western blot analysis showed that AA147 and AA263 increased total protein levels of both γ2 subunit variants (Figure S5E and S5F).

**Fig. 4:**
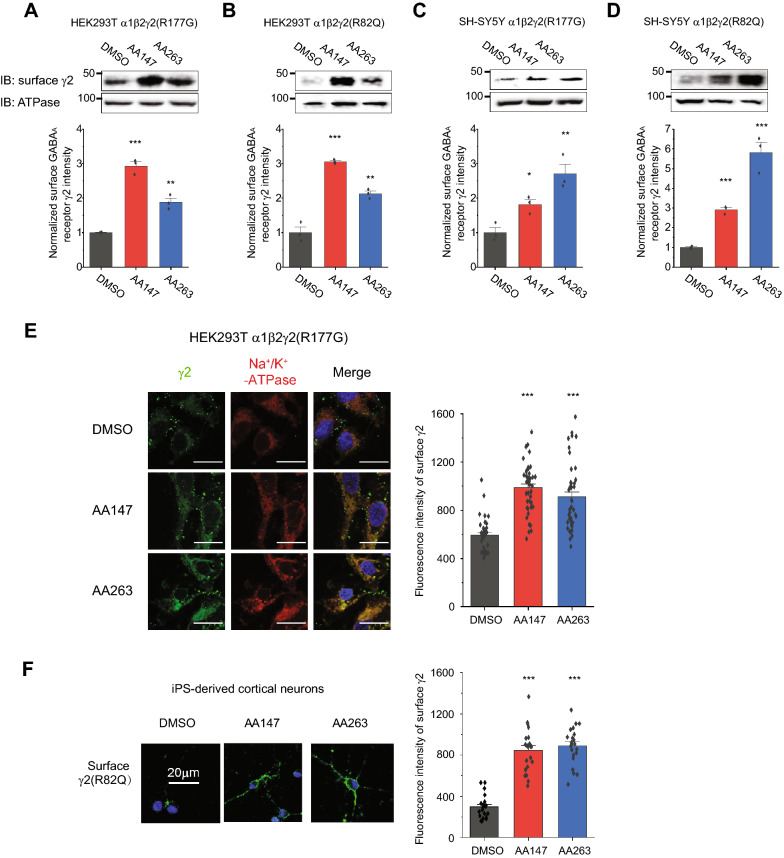
Both AA147 and AA263 promote the surface expression of a variety of trafficking-deficient mutant GABA_A_ receptors. AA147 (5 µM, 24 h) and AA263 (5 µM, 24 h) increase the surface protein expression of the variant γ2 subunits in HEK293T cells stably expressing α1β2γ2(R177G) (**A**) and α1β2γ2(R82Q) (**B**) GABA_A_ receptors. AA147 (2.5 µM, 24 h) and AA263 (2.5 µM, 24 h) increase the surface protein expression of the variant γ2 subunit in neuronal SH-SY5Y cells expressing α1β2γ2(R177G) (**C**) and α1β2γ2(R82Q) (**D**) GABA_A_ receptors according to surface biotinylation analysis. Na^+^/K^+^-ATPase serves as membrane protein loading control. Quantification of the band intensities is shown on the bottom panels (n = 3). **E** HEK293T cells stably expressing α1β2γ2(R177G) receptors were treated with DMSO vehicle control, AA147 (5 µM, 24 h) or AA263 (5 µM, 24 h). Surface γ2 staining was in green (column 1), and plasma membrane marker Na^+^/K^+^-ATPase staining in red (column 2). Merge of these two signals and nucleus staining by DAPI in blue was shown in column 3. Scale bar = 20 μm. Quantification of the fluorescence intensity of the surface subunits from 35–45 cells per condition is shown on the right. **F** Human-induced pluripotent stem cells (hiPS)-derived cortical neurons carrying the γ2(R82Q) variant were treated with DMSO vehicle control, AA147 (2.5 µM, 24 h) or AA263 (2.5 µM, 24 h). Surface γ2 staining was in green and nucleus staining by DAPI was in blue. Quantification of the fluorescence intensity of the surface subunits from 20–30 cells per condition is shown on the right. Each data point is reported as mean ± SEM. One-way ANOVA followed by post-hoc Tukey test was used for statistical analysis. *, *p* < 0.05; **, *p* < 0.01; ***, *p* < 0.001

Surface biotinylation assays showed that AA147 and AA263 increased the surface expression of γ2(R177G) (Fig. 4A) and γ2(R82Q) (Fig. 4B) in HEK293T cells stably expressing the variants. In addition, both AA147 and AA263 enhanced the surface expression of γ2(R177G) (Fig. 4C) and γ2(R82Q) (Fig. 4D) in neuronal SH-SY5Y cells stably expressing the variants. We then performed indirect immunofluorescence microscopy experiments to determine how drug treatments affected the surface staining of variant subunits. Consistently, confocal microscopy experiments demonstrated that AA147 and AA263 increased the surface staining of γ2(R177G), which merged well with the staining of a plasma membrane marker Na^+^/K^+^-ATPase (Fig. 4E) in HEK293T cells expressing the variant. Furthermore, to examine the drug effect in a native-like neuronal environment, we used human-induced pluripotent stem cells (hiPS)-derived cortical neurons expressing the γ2(R82Q) variant and demonstrated that AA147 and AA263 dramatically increased the surface staining of the γ2(R82Q) variant (Fig. 4F).

### ATF6 activating compounds restore synaptic current activity of a variety of trafficking-deficient variant GABA_A_ receptors

**Fig. 5:**
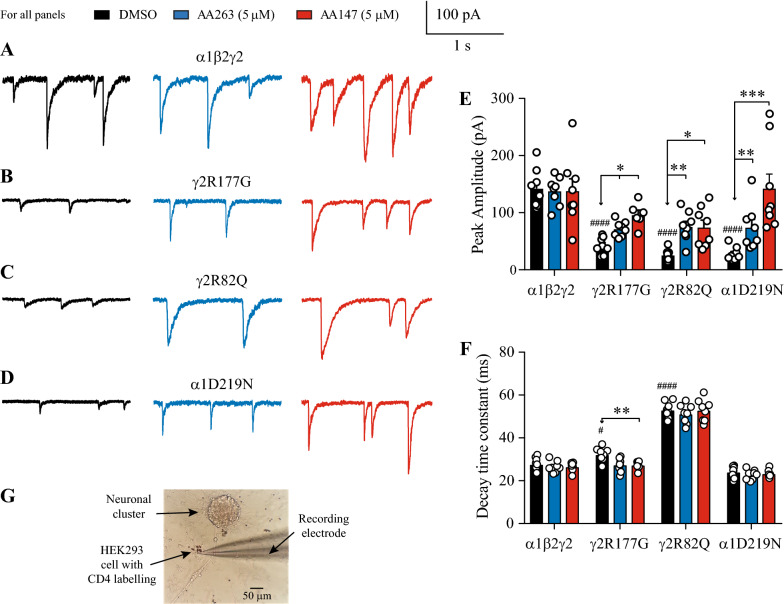
ATF6 activators-induced changes to sIPSC peak current and decay times. **A** sIPSCs mediated by wild-type α1β2γ2 GABA_A_ receptors. **B** sIPSCs mediated by cells transfected with α1, β2 and γ2(R177G) subunits. **C** sIPSCs mediated by cells transfected with α1, β2 and γ2(R82Q) subunits. **D** sIPSCs mediated by cells transfected with α1(D219D), β2 and γ2 subunits. **E** Group bar plots showing changes in sIPSC peak amplitude for the indicated GABA_A_ receptors. **F** Group bar plots showing changes in sIPSC decay times for the indicated GABA_A_ receptors. For all panels, cells were incubated with DMSO (black, left), AA263 (5 μM, 24 h, blue, middle), or AA147 (5 μM, 24 h, red, right). Event frequency was ~ 0.2 Hz in control and drug treated cells. **G** Image of co-culture showing a cluster of primary neurons, axonal extensions and a nearby recorded HEK293 cell that is labelled with CD4 antibody beads. Asterisks represent *p* values for the *post-hoc* comparisons of a two-way ANOVA with and without AFT6 activators exposure, where * *p* < 0.05, ** *p* < 0.01, *** *p* < 0.005. Number signs represent the *p* values for a one-way ANOVA without ATF6 activators exposure, where # *p* < 0.05 and #### *p* < 0.0001. ANOVAs are always compared to α1β2γ2 GABA_A_ receptors

The above data demonstrated that AA147 and AA263 increase the surface trafficking of epilepsy-associated GABA_A_ receptor subunit variants that are prone to ERAD. We hypothesized that seizure activity arises from excessive ERAD that results in impaired inhibitory synaptic input to target neurons. To test this, we profiled the effects that subunit variants and ATF6 activators had on synaptic activity. These experiments were performed using an engineered synaptic co-culture comprising rodent primary neurons and transfected HEK293 cells. This enabled us to record spontaneous synaptic activity from HEK293 cells expressing receptors of defined subunit composition in response to physiological neurotransmitter release [34–36]. Spontaneous inhibitory post-synaptic currents (sIPSCs) mediated by wild-type α1β2γ2L GABA_A_ receptors were recorded in co-cultures that were not incubated in ATF6 activators (drug-naïve controls) or after incubation with either AA263 or AA147 at 5 μM concentration for 24 h (Fig. 5A). Wild-type sIPSC peaks were unaffected by either ATF6 activator, having means of − 138 ± 9 pA (n = 8) from cells incubated with AA263 and − 138 ± 21 pA (n = 8) from cells incubated with AA147 compared to − 143 ± 12 pA from drug-naïve cells (n = 16) (Fig. 5A, E). Similarly, sIPSC decay times recorded from drug-naïve cells were indistinguishable from those recorded from cells that were incubated with either ATF6 activator. The mean decay time measured from drug-naïve cells was 27.8 ± 1.1 ms (n = 16), for cells incubated with AA263 was 26.5 ± 0.5 ms (n = 8), and for cells incubated with AA147 the decay time was 26.4 ± 0.7 ms (n = 8) (Fig. 5A, F). Our sIPSC data suggest that incubation with ATF6 activators has no effect on the key sIPSC parameters of peak and decay time.

Similar experiments were done on receptors containing the variants, γ2L(R177G) (Fig. 5B), γ2L(R82Q) (Fig. 5C) and α1(D219N) (Fig. 5D). Drug naïve controls showed that variant-containing receptors expressed markedly reduced sIPSC peaks compared to wild-type receptors, consistent with our Western blot analysis. The α1β2γ2L(R177G) receptors produced a mean peak of − 42 ± 5 pA (n = 16), the α1β2γ2L(R82Q) receptors had a mean peak of − 26 ± 3 pA (n = 16) and the α1(D219N)β2γ2L had a mean peak of − 28 ± 7 pA (n = 16) (Fig. 5E). An ANOVA test confirmed that all three variant-containing receptors had reduced peak currents (*p* < 0.001 for all three variants). We also compared mean decay times of the wild-type and the three variants. sIPSCs mediated by α1β2γ2L(R177G) receptors decayed with a mean decay time constant of 32.3 ± 1.1 ms, the mean decay time of α1β2γ2L(R82Q) receptors was 53.1 ± 1.3 ms and the mean decay time of α1(D219N)β2γ2L receptors decayed with a mean time constant of 24.2 ± 0.8 ms (Fig. 5F). An ANOVA analysis showed that the decay times for γ2L(R177G)- and γ2L(R82Q)-containing receptors were slower than wild-type (*p* < 0.05 for the γ2L(R177G) and *p* < 0.001 for the γ2L(R82Q) variants). The D219N mutation has faster decay kinetics, which is consistent with previous report [8]. The D219 residue interacts with K247 in the α1 subunits (Additional file 1: Figure S1B), which is in the linker region between the extracellular domain and the transmembrane domain 1 and potentially plays an important role in the transduction of ligand binding to channel gating. Therefore, the D219N mutation could attenuate such an interaction to influence the gating kinetics.

Incubating cells with ATF6 activators had notable effects on sIPSC kinetics, particularly the peak currents. sIPSC peaks increased for all variant-containing receptors (Fig. 5E) and the decay times decreased for the α1β2γ2L(R177G) receptors (Fig. 5F). Peak currents for the α1β2γ2L(R177G) receptors increased to − 72 ± 5 pA (n = 8) when incubated in AA263 and to − 96 ± 9 pA (n = 8) when incubated in AA147. AA263 and AA147 incubation also increased peak currents mediated by α1β2γ2L(R82Q) receptors to − 76 ± 9 pA (n = 8) and − 75 ± 12 pA (n = 8), respectively. The most dramatic increases in peak currents were seen in the α1(D219N)β2γ2L receptors, which increased to − 74 ± 13 pA (n = 8) with AA263 and to − 142 ± 27 pA (n = 8) with AA147, which is close to the wild-type level.

Figure 5F summarizes the data for synaptic decay times. At α1β2γ2L(R177G) receptors, AA147 slightly accelerated the decay times to 27.2 ± 0.9 ms (ANOVA, *p* < 0.01). AA263 incubation resulted in a mean decay time of 27.3 ± 1.1 ms, which did not reach the significance threshold. No change in decay time was detected for the α1β2γ2L(R82Q) receptors. AA263 and AA147 incubation produced decay times of 51.1 ± 1.6 ms and 52.9 ± 1.8 ms, respectively. Similarly, incubation with either activator did not affect the decay times of sIPSCs mediated by the α1(D219N)β2γ2L receptors, which were 22.7 ± 0.8 ms and 23.1 ± 0.6 ms for AA263 and AA147, respectively.

Our data are consistent with previously reported sIPSC data using the same engineered co-culture method [15] and demonstrate the ATF6 activators restore sIPSCs in GABA_A_ receptors that contain variant subunits that are prone to excessive ERAD. Two pieces of evidence suggest that ATF6 activators increase the surface trafficking of the variant subunits and their incorporation into triheteromeric GABA_A_ receptors. Firstly, drug treatment not only increased the peak currents mediated by α1β2γ2L(R177G) receptors, but it also resulted in a decrease in the decay times (Fig. 5E, F) towards that of wild-type receptors. The latter observation suggests the incorporation of the γ2 subunit [15, 36]. Secondly, the increase in peak currents mediated by α1(D219N)β2γ2L receptors also suggests that drug treatment increased the trafficking of the variant subunit because the α1 subunit is required for the formation of functional receptors [36, 37].

### ATF6 activators produce sIPSCs that exhibit triheteromeric receptor pharmacology

The functional analysis of sIPSCs suggests that ATF6 activators increase synaptic currents by enhancing the surface trafficking of the ERAD prone variant subunit. Further, we sought additional evidence for this inference using pharmacology, particularly for the two γ2L variants. We used diazepam (DZP), which targets a high-affinity binding site formed at the α-γ interface [38, 39] and Zn^2+^ ions that inhibit diheteromeric GABA_A_ receptors comprising only α and β subunits with greater potency than triheteromeric αβγ GABA_A_ receptors [40].

**Fig. 6:**
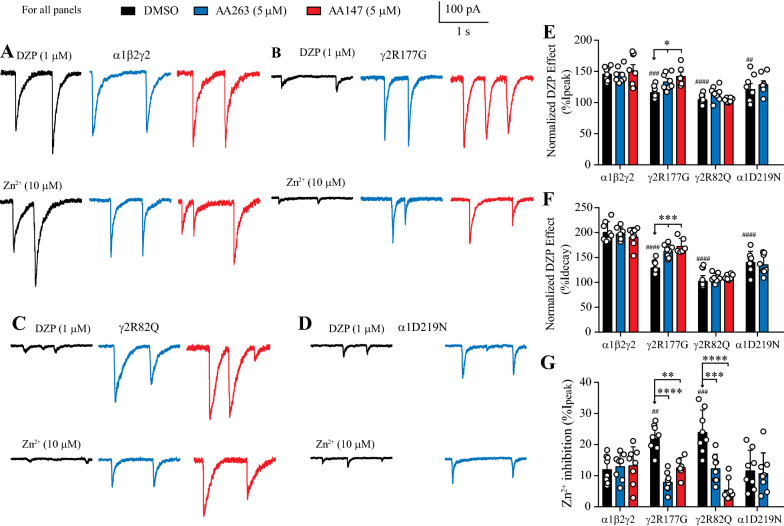
ATF6 activators-induced changes in sIPSC pharmacology. **A** sIPSCs mediated by wild-type α1β2γ2 GABA_A_ receptors. **B** sIPSCs mediated by cells transfected with α1, β2 and γ2(R177G) subunits. (**C**) sIPSCs mediated by cells transfected with α1, β2 and γ2(R82Q) subunits. **D** sIPSCs mediated by cells transfected with α1(D219N), β2 and γ2 subunits. **E** Group bar plots showing the effect of DZP treatment on sIPSC peak amplitude for the indicated GABA_A_ receptors. Normalized DZP effect (%I_peak_) = I_peak_ (DZP) / I_peak_ (control) * 100. The control I_peak_ values were obtained from Fig. 5E. F Group bar plots showing the effect of DZP enhancement on sIPSC decay times for the indicated GABA_A_ receptors. Normalized DZP effect (%I_decay_) = decay time constant (DZP) / decay time constant (control) * 100. The control decay time constant values were obtained from Fig. 5F. G Group bar plots showing Zn^2+^ inhibition of sIPSC peak amplitude for the indicated GABA_A_ receptors. Zn^2+^ inhibition (%I_peak_) = (I_peak_ (control)—I_peak_ (Zn^2+^)) / I_peak_ (control) * 100. The control I_peak_ values were obtained from Fig. 5E. For all panels, cells were incubated with DMSO (black, left), AA263 (5 μM, 24 h, blue, middle), or AA147 (5 μM, 24 h, red, right). Event frequency was ~ 0.2 Hz in control and drug treated cells. Asterisks represent *p* values for the post-hoc comparisons of a two-way ANOVA with and without AFT6 activators exposure, where * *p* < 0.05, ** *p* < 0.01, *** *p* < 0.005, **** *p* < 0.001. Number signs represent the *p* values for a one-way ANOVA without ATF6 activators exposure, where ^##^*p* < 0.01, ^###^*p* < 0.005 and ^####^*p* < 0.0001. ANOVAs are always compared to α1β2γ2 GABA_A_ receptors

Continuous perfusion of 1 μM DZP onto recorded cells expressing wild-type receptors resulted in a marked increase in sIPSC peak (to 147 ± 4%) and mean decay time (202 ± 7%) (n = 16, Fig. 6A top, E and F). A similar experiment on wild-type receptors demonstrated 10 μM Zn^2+^ blocked peak currents by only 12 ± 2% (n = 16, Fig. 6A bottom, 6G). By contrast, the same pharmacological testing on cells transfected only with α1 and β2 subunits showed no effect by DZP on peak (100 ± 4%) or decay (102 ± 3%) and 83 ± 2% inhibition by Zn^2+^ (n = 8, Additional file 1: Figure S6). These data show that when cells were transfected with α1, β2 and γ2L subunit cDNAs the resultant wild-type receptors contained the γ subunit [15]. These pharmacological tests were used as references for changes in γ subunit incorporation in response to ATF6 activators in wild-type and variant-containing GABA_A_ receptors. AA263 and AA147 incubation had no additional effect on sIPSCs mediated wild-type receptors that were perfused with DZP. Peak currents were potentiated to 144 ± 4% and the mean decay time by 198 ± 4% when incubated by AA263, whereas the AA147 potentiated peak currents by 152 ± 9% and the mean decay time by 192 ± 6%. Exposure to 10 μM Zn^2+^ inhibited currents by 13 ± 1% in AA263 treated cells and 13 ± 2% with AA147 treatment.

At α1β2γ2L(R177G) receptors (Fig. 6B), DZP perfusion potentiated sIPSCs by increasing peaks by 117 ± 3% and decay times by 131 ± 4% in ATF6 activator naïve controls. AA263 incubation further increased peak currents by 143 ± 6% and decay times by 173 ± 6%, whereas AA147 treatment increased peak and decay times to 134 ± 5% and 163 ± 5%, respectively (Fig. 6B, E and F). From a control level of Zn^2+^ induced inhibition of 22 ± 2%, AA263 incubation reduced this to 13 ± 1%, whereas AA147 reduced it to 8 ± 1% (Fig. 6B and G). These results are consistent with an increase in γ(R177G) incorporation into triheteromeric GABA_A_ receptors and reveal that treatment with these ATF6 activators enhances the trafficking of the affected variant subunit. The α1β2γ2L(R82Q) receptors exhibited insensitivity to DZP in control conditions and after treatment with either AA147 or AA263. DZP perfusion produced peak currents that were unchanged at 105 ± 3% in drug naïve cells. After AA263 and AA147 incubation, DZP remained ineffective at altering peak currents, which were 104 ± 1% and 115 ± 4% compared to sIPSCs controls. The decay times were similarly unaffected by DZP. In drug naïve controls DZP produced a mean decay time that was 104 ± 3% compared to 109 ± 3% after AA263 and 110 ± 2% after AA147 incubation. The insensitivity to DZP exhibited by α1β2γ2L(R82Q) receptors was similar to that seen in diheteromeric α1β2 GABA_A_ receptors (Additional file 1: Figure S6). However, perfusion with Zn^2+^ produced data that strongly suggested that treatment with AA147 and AA263 did indeed increase the synthesis and subsequent incorporation of the variant, γ2L(R82Q) subunit into triheteromeric receptors. Drug naïve cells transfected with α1, β2 and γ2L(R82Q) subunits were inhibited by 24 ± 2%, which was significantly less than that observed for wild-type α1β2γ2L receptors (12 ± 2%, p < 0.001). After incubation with AA263 the extent of inhibition by Zn^2+^ at α1β2γ2L(R82Q) receptors was 12 ± 2% and with AA147 was 6 ± 1% (Fig. 6C, G).

Receptors containing the α1(D219N) variant demonstrated clear increases in peak currents and an unaffected mean decay time in compound treated cells (Fig. 5). These data indicate that AA147 and AA263 promoted α1(D219N) incorporation into triheteromeric receptors. However, we also tested DZP and Zn^2+^ on AA263 treated cells as an additional control to wild-type receptors. DZP potentiation of α1(D219N)β2γ2L receptor peak currents was less pronounced (122 ± 7%) compared to wild-type, as was the mean decay time (142 ± 7%), (Fig. 6D, E). AA263 treatment resulted in a potentiation of peak and decay time by DZP of 130 ± 5% and 137 ± 75, respectively. These data suggest that even though the surface expression of the variant subunit is enhanced by compound treatment, the α1(D219N) variant produces alterations to DZP sensitivity that are intrinsic to the receptors, as previously noted [15]. As predicted for γ2-containng receptors, Zn^2+^ was relatively insensitive at α1(D219N)β2γ2L receptor, inhibiting sIPSCs in drug naïve cells to 12 ± 2% and 11 ± 2% after AA263 incubation (Fig. 6D, G).

In summary, for γ2(R177G) and γ2(R82Q)-containing receptors, AA147 and AA263 treatment led to reduced Zn^2+^ sensitivity (Fig. 6G), consistent with the notion that these ATF6 activators promoted the incorporation of these variants into functional triheteromeric αβγ receptors. The α1(D219N)-containing receptors exhibit αβγ-like (wild-type) Zn^2+^ sensitivity (Fig. 6G) and an increase in peak current potentiation that approaches wild-type levels (Fig. 5E).

## Discussion

**Fig. 7:**
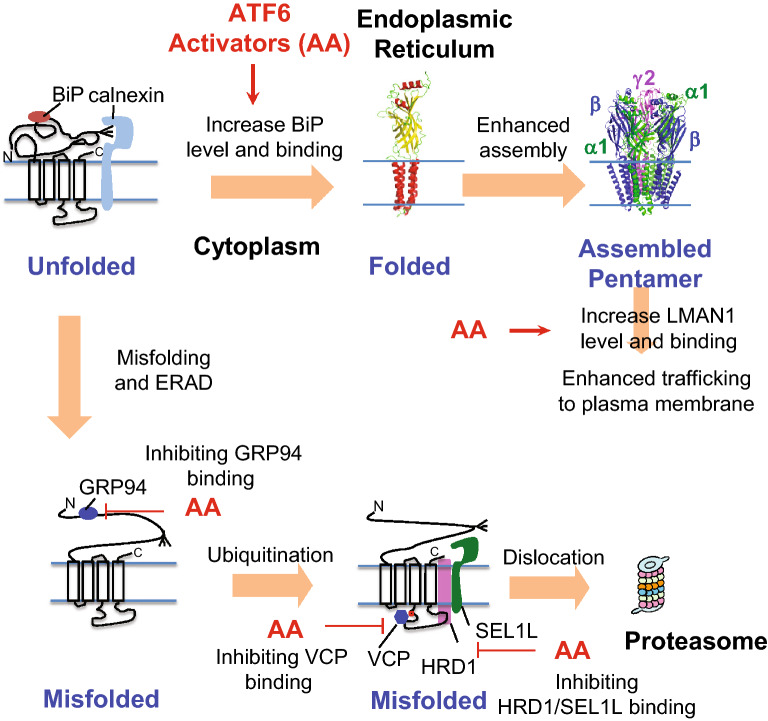
Mechanism of action of AA147 and AA263 on GABA_A_ receptor proteostasis. AA147 and AA263 activate the ATF6 pathway to adapt the proteostasis network. They promote the folding of variant subunits by increasing BiP chaperone protein level and binding and enhance their forward trafficking by increasing LMAN1 protein level and binding. In addition, they attenuate the ERAD of variant subunits by inhibiting GRP94-HRD1/VCP-mediated degradation pathway. As a result, pharmacological activation of ATF6 increases the functional surface expression of variant GABA_A_ receptors

Our data unambiguously corroborates that the ATF6 activators AA147 and AA263 enhanced the folding and reduced the degradation of the epilepsy-causing variant subunits, leading to their productive trafficking to the Golgi and onward to the plasma membrane. Once they reach the plasma membrane, the function of the variant receptors is restored, in some cases to wild-type levels, as shown by our synaptic current data. Our results support the mechanism for AA147 and AA263 mediated rescue of misfolding-prone variant GABA_A_ receptors shown in Fig. 7. AA147 and AA263 activate the ATF6 pathway, which adjusts the ER proteostasis network. Both AA147 and AA263 increase the BiP protein levels and the interaction between BiP and the variant subunits, which enhances the productive folding and assembly of pentameric receptors in the ER. Moreover, to enhance the forward trafficking from the ER to the Golgi, they increase the protein level of LMAN1, an important trafficking receptor for GABA_A_ receptors. In addition, AA147 and AA263 reduce the ERAD of pathogenic receptors. To achieve this, they reduce the interaction between variant GABA_A_ receptors and salient ERAD factors, including GRP94 in the ER lumen, HRD1 on the ER membrane, and VCP in the cytosol. It is the orchestration of the ER proteostasis network after the treatment with AA147 and AA263 that promote the folding, trafficking, and function of the pathogenic GABA_A_ receptors.

GABA_A_ receptors are major therapeutic targets for epilepsy treatment [41]. Our discovery that small molecule proteostasis regulators restore variant receptor trafficking defects represents a potential new class of medications for epilepsy, particularly for pathogenic GABA_A_ receptors. AA147, in particular, is a suitable candidate because it readily crosses the blood–brain barrier [26]. By promoting the surface expression of trafficking-deficient variant receptors to synaptic sites, proteostasis regulators have dual roles. Firstly, they restore a degree of synaptic input; secondly, they make the variant receptors available to conventional anti-seizure drugs that either affect the receptors directly on the surface, such as benzodiazepines [41] or potentiate GABAergic inhibitory input by releasing GABA the neurotransmitter, such as valproate [42]. The current *trial-and-error* approach for treating neurological disorders associated with post-synaptic receptor variants is prone to treatment failure in about one-in-three, and displays highly variable success in the remaining two-thirds of affected individuals [43]. One of the reasons such a high proportion of affected individuals are drug-resistant could be that trafficking defects in gene variants is a major pathological mechanism.

Our study highlights the relevance of profiling receptor variants for trafficking deficits and functional deficits that are intrinsic to the receptors, and explores targeted treatment options that are selective for specific classes of variants. There are emerging examples of successful cases of personalized therapy involving variant ion channel genes [43–46], including genes that encode excitatory neurotransmitter-gated synaptic receptors [47, 48]. However, there are no cases of personalized medicine that target the inhibitory GABA_A_ receptor, even though it is a major cause of neurological disorders and a major therapeutic target for epilepsy and other neurological indications.

There are many established mouse models for epilepsy, which target different epilepsy-causing genes [49]. Due to the advance of genome editing technology, during the last several years, several epilepsy mouse models carrying GABA_A_ receptor variants, such as A322D in the α1 subunit, D120N in the β3 subunit, and Q390X in the γ2 subunit, have been generated [50–52]. For example, the *GABRB3* D120N knock-in mouse model was reported as the first genetic model for Lennox-Gastaut Syndrom, a severe form of childhood epilepsy, in 2020, and the knockin mice displayed spontaneous seizures, impaired learning and memory, and increased anxiety [52]. However, in the knockin mice, the protein expression levels and membrane trafficking of the major α1, β3, and γ2 subunits of GABA_A_ receptors were not changed compared to those in wild-type mice. The *GABRA1* A322D knock-in mouse model recapitulated the absence seizure phenotype associated with juvenile myoclonic epilepsy, and molecular mechanism study showed that the A322D mutation in the transmembrane helix 3 reduced the α1 subunit protein level as well as the miniature IPSCs in motor cortex [50]. The *GABRG2* Q390X knock-in mice displayed spontaneous seizure and neurobehavioral comorbidities that were observed in Dravet Syndrome, and molecular mechanism study demonstrated that the Q390X mutation, which terminated the subunit before the transmembrane helix 4, caused the aggregation of the variant and reduced miniature IPSCs [51]. It would be of great interest to evaluate the effect of proteostasis regulators [15, 16], including ATF6 activators, on correcting seizure phenotypes in proper mouse models. For example, AA147 is promising because it readily crosses the blood–brain barrier, and intravenous injection of AA147 at 2 mg/kg was well tolerated in mice and provided protective effects in renal and cerebral ischemia/reperfusion model [26]. Further, medicinal chemistry could be performed to improve the potency and other desired pharmacological properties of AA147 and other proteostasis regulators.

AA147 has been shown to rescue aggregation-prone proteins by targeting them for more effective clearance [53]. In this study, we demonstrate that the ATF6 activators AA147 and AA263 restore trafficking deficits and synaptic function of neurotransmitter-gated ion channels containing variant GABA_A_ receptor subunits. This indicates that pharmacological activation of the ATF6 pathway can adapt the proteostasis network with subtle differences in different pathogenic conditions, aiming to optimal stress reduction and functional rescue. Ultimately, this work highlights the potential for targeting ER proteostasis, and more specifically UPR pathways, to enhance the folding, assembly, and trafficking of disease-relevant mutant receptors. Our work suggests that similar approaches can be used more broadly to improve surface expression of mutant receptors associated with other types of protein misfolding diseases.

## METHODS

### Reagents

AA147 and AA263 were described in previous publications [23]. Ceapin-A7 (#SML2330) and apyrase (#A6237) were obtained from Sigma. PF429242 (#15,140) and 4μ8c (#22,110) were obtained from Cayman Chemical. DMSO (0.1% v/v final concentration) was used as a vehicle control, which matches its concentration in AA147 and AA263.

### Plasmids

The pCMV6 plasmids containing human GABA_A_ receptor α1 (Uniprot no. P14867-1), β2 (isoform 2, Uniprot no. P47870-1), γ2 (isoform 2, Uniprot no. P18507-2) subunits, and pCMV6 Entry Vector plasmid (pCMV6-EV) were obtained from Origene. The human GABA_A_ receptor α1 subunit missense mutation D219N and human GABA_A_ receptor γ2 subunit missense mutation R82Q or R177G were constructed using QuikChange II site-directed mutagenesis Kit (Agilent Genomics). The FLAG tag was inserted between Leu31 and Gln32 in the α1 subunit, and inserted between Asn28 and Asp29 in the β2 subunit, which do not influence their trafficking [54]. All cDNA sequences were confirmed by DNA sequencing.

### Antibodies

The mouse monoclonal anti-α1 subunit antibody (clone BD24) (#MAB339) and rabbit polyclonal anti-γ2 subunit antibody (#AB5559) were obtained from Millipore. The rabbit polyclonal anti-γ2 antibody was also obtained from Synaptic Systems (#224,003). The mouse monoclonal anti-β-actin (#A1978) was obtained from Sigma Aldrich. The rabbit polyclonal anti-calnexin (CANX) (#ADI-SPA-860-F) and rat monoclonal anti-GRP94 (clone 9G10) (#ADI-SPA-850-F) antibodies were purchased from Enzo Life Sciences. The goat polyclonal anti-SEL1L antibody (#PA5-18,943) was obtained from ThermoFisher Scientific. The rabbit polyclonal anti-HRD1 antibody (#AP2184e) and rabbit polyclonal anti-BiP antibody (#AP5041C) were obtained from Abgent. The rabbit monoclonal anti-VCP (#ab109240), rabbit monoclonal anti-LMAN1 (#ab125006), and rabbit monoclonal anti-Na^+^/K^+^-ATPase (#ab76020) antibodies were from Abcam. The mouse monoclonal anti-Na^+^/K^+^-ATPase (#sc-48345), a plasma membrane marker, was obtained from Santa Cruz Biotechnology. The rabbit monoclonal anti-XBP1s antibody (#12,782) was purchased from Cell Signaling.

### Cell culture and transfection

HEK293T cells (#CRL-3216, donor sex: female) and SH-SY5Y cells (#CRL-2266, donor sex: female) were obtained from ATCC. Cells were cultured in Dulbecco’s Modified Eagle Medium (DMEM) with 10% heat-inactivated fetal bovine serum (FBS) and 1% Penicillin–Streptomycin at 37 °C in 5% CO_2_.

Stable cell lines expressing α1β2γ2, α1(D219N)β2γ2, α1β2γ2(R177G), or α1β2γ2(R82Q) GABA_A_ receptors were generated using the G418 selection method. Cells were grown in a 6-well plate and reached ~ 70% confluency. Cells were then transfected with α1:β2:γ2 (0.3 μg:0.3 μg:0.3 μg), α1(D219N):β2:γ2 (0.3 μg:0.3 μg:0.3 μg), α1:β2:γ2(R177G) (0.3 μg:0.3 μg:0.3 μg), or α1:β2:γ2(R82Q) (0.3 μg:0.3 μg:0.3 μg) plasmids using TransIT-2020 (Mirus, #MIR5400) according to the manufacturer’s instruction. Forty-eight hours post transfection, cells were passaged to 10 cm dishes and selected in DMEM with 10% FBS and 1% Penicillin–Streptomycin supplemented with 1 mg/mL G418 (Enzo Life Sciences, #ALX-380–013) for 15 days. Cells were then maintained in media containing 0.4 mg/mL G418. The G418 resistant polyclonal cells expressing GABA_A_ receptor variants, which were verified by Western blot analysis, were used for experiments.

### Western blot analysis

Cells were harvested with Trysin and lysed with lysis buffer (50 mM Tris, pH 7.5, 150 mM NaCl, and 2 mM DDM) supplemented with complete protease inhibitor cocktail (Roche #04,693,116,001). Lysates were cleared by centrifugation (21,000×*g*, 10 min, 4 °C). Protein concentrations were measured by MicroBCA assay (ThermoFisher Pierce #23,235). Aliquots of cell lysates were loaded with 4 × Laemmli buffer (Biorad #161–0747) with 10% 2-Mercaptoethanol (Sigma #M3148) and separated in an 8% SDS-PAGE gel. Western blot analysis was performed using appropriate antibodies. The β-actin serves as a total protein loading control, whereas Na^+^/K^+^-ATPase serves as a plasma membrane protein loading control. If proteins of interest have similar molecular weight with β-actin, such proteins were detected first; afterwards, the blots were stripped and re-probed with β-actin. Band intensity was quantified using Image J software from the NIH. For quantification, the total protein was first normalized to β-actin and then the DMSO vehicle control, whereas the surface protein was first normalized to Na^+^/K^+^-ATPase and then the DMSO vehicle control.

### Quantitative Reverse transcription (RT)-PCR

The relative expression levels of target genes were analyzed using quantitative RT-PCR according to published procedure [14]. Briefly, cells were treated with compounds for the indicated amount of time before total RNA was extracted from the cells using RNeasy Mini Kit (Qiagen #74,104). cDNA was synthesized from 500 ng of total RNA using QuantiTect Reverse Transcription Kit (Qiagen #205,311). Quantitative PCR reactions (45 cycles of 15 s at 95 °C, 15 s at 59 °C, and 60 s at 72 °C) were performed using cDNA, PowerUp SYBR Green Master Mix (Applied Biosystems #A25776) and corresponding primers in the QuantStudio 3 Real-Time PCR System (Applied Biosystems) and analyzed using QuantStudio software (Applied Biosystems). The forward and reverse primers for *GABRA1* are 5′-GTCACCAGTTTCGGACCCG-3′ and 5′-AACCGGAGGACTGTCATAGGT-3′; the forward and reverse primers for *RPLP2* (housekeeping gene control) are 5′-CGTCGCCTCCTACCTGCT-3′ and 5′-CCATTCAGCTCACTGATAACCTTG-3′. Threshold cycle (C_T_) was extracted from the PCR amplification plot, and the ΔC_T_ value was defined as: ΔC_T_ = C_T_ (target gene)—C_T_ (housekeeping gene). The relative mRNA expression level of target genes of drug-treated cells was normalized to that of untreated cells: Relative mRNA expression level = 2 exp [- (ΔC_T_ (treated cells)—ΔC_T_ (untreated cells))]. Each data point was evaluated in triplicates.

### Biotinylation of cell surface proteins

HEK293T cells or SH-SY5Y cells stably expressing GABA_A_ receptor variants were plated in 6-cm dishes for surface biotinylation experiments according to published procedure [8]. Briefly, intact cells were washed with ice-cold PBS and incubated with the membrane-impermeable biotinylation reagent Sulfo-NHS SS-Biotin (1 mg ⁄ mL; APExBIO, #A8005) in PBS containing 0.1 mM CaCl_2_ and 1 mM MgCl_2_ (PBS + CM) for 30 min at 4 °C to label surface membrane proteins. To quench the reaction, cells were incubated with 10 mM glycine in ice-cold PBS + CM twice for 5 min at 4 °C. Sulfhydryl groups were blocked by incubating the cells with 5 nM N-ethylmaleimide (NEM) in PBS for 15 min at room temperature. Cells were solubilized for 1 h at 4 °C in lysis buffer (DDM, 2 mM; Tris–HCl, 50 mM; NaCl, 150 mM; and EDTA, 5 mM; pH 7.5) supplemented with Roche complete protease inhibitor cocktail and 5 mM NEM. The lysates were cleared by centrifugation (21,000 × g, 10 min at 4 °C) to pellet cellular debris. The supernatant contained the biotinylated surface proteins. Biotinylated surface proteins were affinity-purified from the above supernatant by incubating for 1 h at 4 °C with 40 μL of immobilized neutravidin-conjugated agarose bead slurry (ThermoFisher Pierce #29,201). The samples were then subjected to centrifugation (21,000 × g, 10 min, at 4 °C). The beads were washed 3 times with buffer (Tris–HCl, 50 mM; NaCl, 150 mM; and EDTA, 5 mM; pH 7.5). Surface proteins were eluted from beads by boiling for 5 min with 30 μL of LSB ⁄ Urea buffer (2 × Laemmli sample buffer (LSB) with 100 mM DTT and 6 M urea; pH 6.8) for SDS-PAGE and Western blotting analysis.

### MTT cell toxicity assay

SH-SY5Y cells were plated in a 96-well plate at 2 × 10^4^ cells per well, incubated at 37 °C overnight, and then treated with AA147 (24 h) or AA263 (24 h). To each well, 500 μg/mL MTT (3-(4, 5-dimethylthiazol-2-yl)-2, 5-diphenyltetrazolium bromide) (from Amresco, 97,062–376) was added, and the plate was incubated at 37 °C for 3 h. Culture media was removed, and the crystals were dissolved by 150 µL of DMSO. The absorbance at 590 nm was measured using a microplate reader (Molecular Device). Cell viability was expressed as the ratio of the signal obtained from AA147 or AA263 treated samples over DMSO control samples, and data were presented as mean±SEM.

### Confocal immunofluorescence

The labeling of surface GABA_A_ receptors and confocal immunofluorescence microscopy analysis were performed according to published procedure [14]. Briefly, cells on coverslips were fixed for 3 min with 4% formaldehyde on ice and incubated in 100 μL of HEPES buffer (HEPES 25 mM, NaCl 140 mM, KCl 5.4 mM, CaCl_2_ 1.8 mM, glucose 15 mM, pH = 7.4) containing mouse monoclonal anti-α1 antibody (Millipore #MAB339) and rabbit monoclonal anti-Na^+^/K^+^-ATPase, a plasma membrane marker (Abcam #ab76020), or rabbit polyclonal anti-γ2 antibody (Synaptic Systems #224,003) and mouse monoclonal anti-Na^+^/K^+^-ATPase, a plasma membrane marker (Santa Cruz Biotechnology #sc-48345) for 1.5 h. The primary antibodies were used at 1:300 dilutions. Cells were then incubated with 100 μL of HEPES buffer containing an Alexa 488-conjugated secondary antibody (1:600) and an Alexa 594-conjugated secondary antibody (1:600). Afterwards, cells were permeabilized with saponin (0.2%) for 10 min and incubated with DAPI (1 µg/mL) for 3 min to stain the nucleus. The coverslips were then mounted and sealed. For confocal immunofluorescence microscopy, an Olympus IX-81 Fluoview FV1000 confocal laser scanning system was used. A 60X objective collected images using FV10-ASW software. Quantification of the fluorescence intensity after background correction was done using the ImageJ software from the NIH.

### Cycloheximide-chase assay

HEK293T cells stably expressing α1(D219N)β2γ2 GABA_A_ receptors were seeded in 6-well plates and incubated at 37 °C overnight. Cells were then treated with AA147 or AA263 for 24 h prior to cycloheximide-chase. To stop protein translation, cells were treated with 100 μg/mL cycloheximide (Enzo Life Sciences # ALX-380–269). Cells were then chased for the indicated time, harvested, and lysed for protein analysis.

### Immunoprecipitation

The cell lysates were incubated with 2.0 µg of mouse anti-α1 antibody (Millipore #MAB339) overnight at 4 °C, and then with 30 µL of protein A/G plus agarose beads overnight at 4 °C. The beads were collected by centrifugation at 8000 × g for 60 s, and washed three times with lysis buffer. The α1 subunit complex was eluted by incubation with 30 µL of SDS loading buffer in the presence of DTT. The immunopurified eluents were separated in 8% SDS-PAGE gel, and Western blot analysis was performed.

### Endoglycosidase H (endo H) enzyme digestion assay

To remove asparaginyl-*N*-acetyl-D-glucosamine in the N-linked glycans incorporated on the α1 subunit in the ER, total cell lysates were digested with Endo H enzyme (NEBiolab #P0702L) with G5 reaction buffer at 37 °C for 1 h. The Peptide-N-Glycosidase F (PNGase F) (NEBiolab, #P0704S) enzyme-treated samples served as a control for unglycosylated α1 subunits. Treated samples were then subjected to western blot analysis.

### Human iPS-derived neuron cultures

Human iPS derived cortical neurons carrying the heterozygous R82Q variant in the γ2 subunit were obtained from FUJIFILM Cellular Dynamics, Inc. (#DDP-NCR-0.5 × 01,279.770). Peripheral blood was obtained from a healthy donor (male) and then reprogrammed into iPS cells using the patented footprint-free episomal method [55]. The nuclease-mediated genome editing technology was employed to introduce the R82Q mutation into the parental iPS cell line. To generate differentiated neurons, iPS cells, which were expanded under defined, feeder-free conditions, were cultured in neuronal differentiation medium for 7 days to induce differentiation, followed by culturing in neuronal maturation medium. The differentiated neurons were cryopreserved after 28–30 days total in culture. The neuronal cells produced using this method are cortical neurons as defined by *DACH1*, *FOXG1* and *OTX2* gene expression. The R82Q mutation in both iPS cells and differentiated GABANeurons has been confirmed using sequence analysis of the gene-edited region. Neurons were plated in poly-D-lysine (Sigma #P6407) and laminin (Sigma #L2020)-coated coverslips (Fisher #12–545-80P) in 24-well plates following the company instruction. Half medium was replaced with neurobasal medium (ThermoFisher #21,103,049) supplemented with 2% B27 (ThermoFisher #17,504,044) and 1% GlutaMAX (ThermoFisher #35,050,061) the next day and then every 3 days. Surface staining for the GABA_A_ γ2 subunit was carried out at day 7 post plating.

### Engineered synapse preparation and electrophysiology

*Cell Culture and Molecular Biology*—Human α1 (pCIS2), β2 (pcDNA3.1 +) and γ2L (pcNDA3.1 +) subunits were transfected at a subunit plasmid ratio of 1α:1β:4γ or 1α:1β: (total DNA was 0.2–2.0 μg), into HEK293AD cells using Ca^2+^ phosphate-DNA coprecipitation. Added to the transfection mixture was the neuroligin splice variant neuroligin 2A, which promoted the formation of synaptic contacts with the neurons [36] and the transfection markers, CD4 or GFP.

Primary neuronal cultures were prepared using standard protocols [36]. The cortices of E18 rat embryos were triturated and plated at ~ 90,000 cells per 12-mm poly-D-lysine-coated coverslip in DMEM with 10% foetal bovine serum. The entire medium was replaced with neurobasal medium that included 2% B27 and 1% GlutaMAX supplements the next day and again after 1 week. Transfected HEK293 cells were introduced to the primary neuronal cultures after 3 − 5 weeks. Synaptic recordings were done 1–3 days later. The primary neurons used on our HEK293-neuronal co-cultures were harvested from 15 sets of rat embryos over a period of 8 months.

*Electrophysiology*—All experiments were performed at room temperature in the whole-cell configuration of the patch clamp technique, at a holding potential of − 70 mV. The intracellular solution was composed of (in mM): 145 CsCl, 2 CaCl_2_, 2 MgCl_2_, 10 HEPES, and 10 EGTA, adjusted to pH 7.4 with CsOH. Cells were continuously perfused with extracellular solution made up of (in mM): 140 NaCl, 5 KCl, 2 CaCl_2_, 1 MgCl_2_, 10 HEPES, and 10 D-glucose, adjusted to pH 7.4 with NaOH. Each experiment consisted of a 10 min recording period is standard extracellular solution (control) followed by perfusion of diazepam (DZP) for a 0.5 min, a wash period for several minutes, and finally a Zn^2+^ perfusion for another 5 min. DZP (1 μM) and Zn^2+^ (10 μM) were applied to the recorded cell directly via parallel plastic tubes. ATF6 activators at 5 μM were added to the co-cultures at the time of introducing the transfected HEK293 cells and left to incubate 24 h prior to the commencement of the experiment.

Spontaneous inhibitory post-synaptic currents (sIPSCs) were recorded using a Axopatch 200B amplifier and pClamp 10 software, filtered (− 3 dB, 4-pole Bessel) at 2 kHz and sampled at 10 kHz. sIPSCs were analysed for peak current and decay times in Axograph X. Between 50 and 800 isolated sIPSCs were selected and averaged from each cell. The current decay phase of the averaged current was fitted to a single exponential to obtain the decay time constant. These averages were then pooled into data sets, from which means were calculated. Recordings with an access resistance of > 12 MΩ were not included in the analysis. Currents containing multiple peaks and extraneous inflections in the decay phase were manually excluded. Bar plots and statistical analysis was done in GraphPad Prism.

### Quantification and statistical analysis

All data are presented as mean ± SEM. Statistical significance was evaluated using two-tailed Student’s *t*-test if two groups were compared and one-way ANOVA followed by post-hoc Tukey test if more than two groups were compared. A *p* < 0.05 was considered statistically significant. Two-way ANOVAs were used to test for statistical significance in sIPSC properties between treated and untreated groups accompanied by Sidak’s post-hoc test from 8 cells per data set.

## Supplementary Information

**Additional file 1.** Additional figures.

## Data Availability

All cell lines, plasmids, and data generated in this study will be provided upon reasonable request after acceptance of the paper for publication.
