# Supplementary material for: Pharmacological activation of ATF6 remodels the proteostasis network to rescue pathogenic GABAA receptors
Source: Cell Biosci. 2022 Apr 27;12:48. doi: 10.1186/s13578-022-00783-w (PMC9044816; doi:10.1186/s13578-022-00783-w)
Supplement: Supplementary file 1 — Additional file 1. Additional figures. [file 13578_2022_783_MOESM1_ESM.pdf]

Supplementary Information

Pharmacological Activation of ATF6 Remodels the Proteostasis Network to Rescue Pathogenic $GABA_A$ receptors

Meng Wang,[1,#] Edmund Cotter,[2,#] Ya-Juan Wang,[1] Xu Fu,[1] Angela L Whittsette,[1]

Joseph W. Lynch[2], R. Luke Wiseman,[3] Jeffery W. Kelly,[3] Angelo Keramidas,[2,*] Ting-Wei Mu[1,*]

[1]Department of Physiology and Biophysics, Case Western Reserve University School of Medicine, 10900 Euclid Ave, Cleveland, Ohio 44106, USA.

[2]Queensland Brain Institute, the University of Queensland, Brisbane, QLD 4072, Australia.

[3]Department of Molecular Medicine, The Scripps Research Institute, 10550 North Torrey Pines Road, La Jolla, California 92037, USA.

[#]These authors contributed equally.

[*]To whom correspondence should be addressed.

Telephone: +1 (216) 368-0750; Fax: +1 (216) 368-5586; E-mail: tingwei.mu@case.edu

Telephone: +61 7 33463330, E-mail: a.keramidas@uq.edu.au

ORCID ID: 0000-0002-6419-9296 (Ting-Wei Mu)

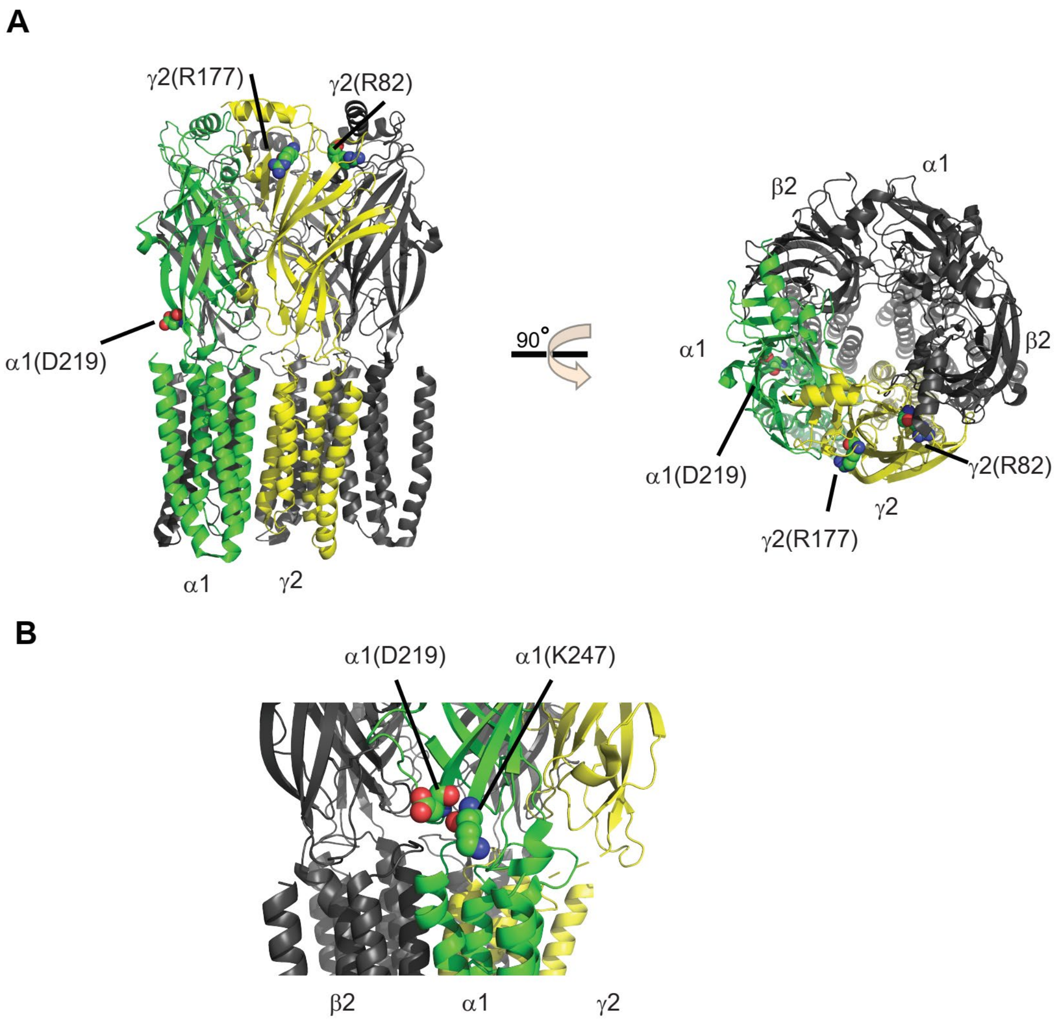

Supplementary Figure S1. **Cartoon representation of the structure of GABA$_A$ receptors.** **(A)** The cryo-EM structure of the α1β2γ2 pentameric GABA$_A$ receptors (6D6U.pdb) was presented using PyMOL. Space filling models were used to show the residue of D219 in one α1 subunit and the residues of R82 and R177 in the γ2 subunit. **(B)** Positions of D219 and K247 in the α1 subunit.

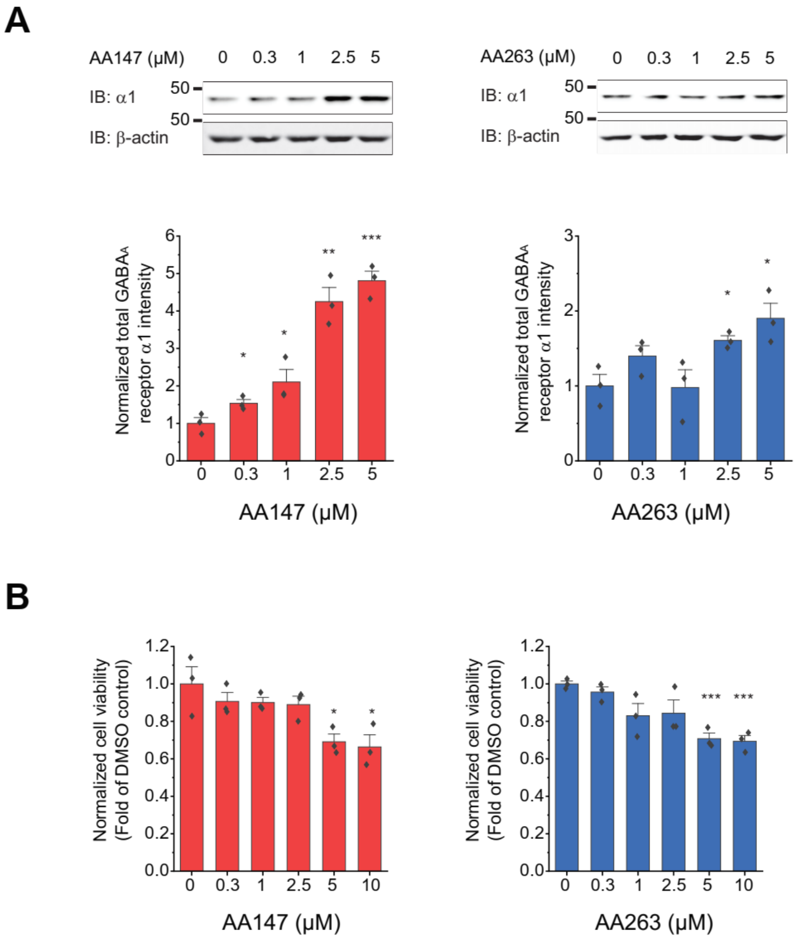

Supplementary Figure S2. **Effect of AA147 and AA263 on neuronal SH-SY5Y cells.** (**A**) Dose-response analysis of AA147 and AA263 treatment (24 h) on the total protein levels of α1(D219N) subunits in human neuronal SH-SY5Y cells stably expressing α1(D219N)β2γ2 $GABA_A$ receptors. β-actin serves as total protein loading control. Quantification of the band intensities is shown on the bottom panels (n = 3). IB: immunoblotting. (**B**) MTT toxicity assay shows the cell viability of SH-SY5Y cells stably expressing α1(D219N)β2γ2 receptors after treatment with varying concentrations of AA147 (24 h) or AA263 (24 h). Each data point is reported as mean ± SEM. One-way ANOVA followed by post-hoc Tukey test was used for statistical analysis. *, $p < 0.05$; **, $p < 0.01$; ***, $p < 0.001$.

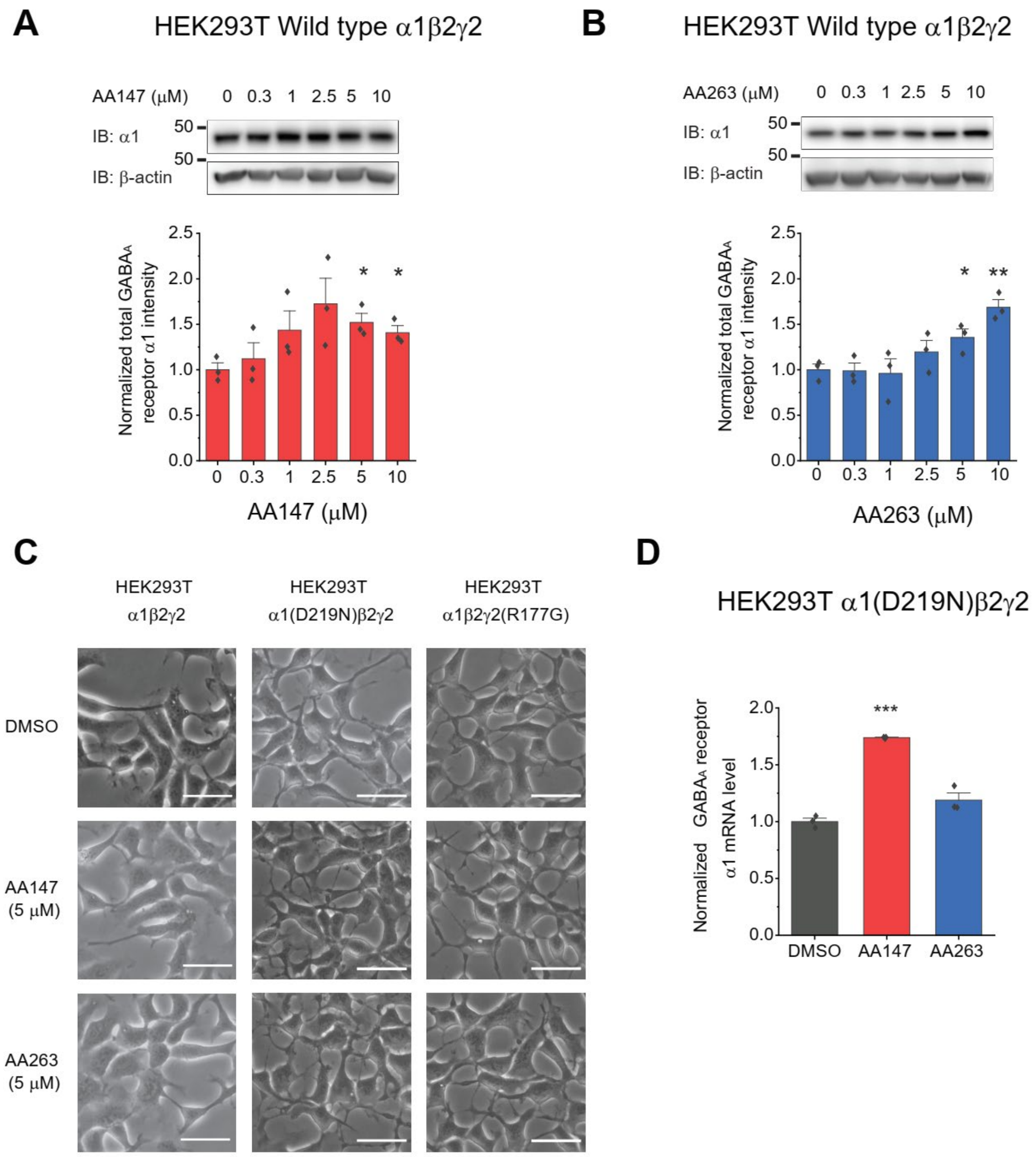

Supplementary Figure S3. **Effect of AA147 and AA263 on the protein and RNA levels of GABA$_A$ receptors and cell morphology in HEK293T cells.** Dose-response analysis of AA147 (**A**) and AA263 treatment (**B**) (24 h) in HEK293T cells expressing α1β2γ2 GABA$_A$ receptors. β-actin serves as total protein loading control. Quantification of the band intensities is shown on the bottom panels (n = 3). IB: immunoblotting. (**C**) Effect of AA147 and AA263 treatment (5 μM, 24 h) on the cell morphology of HEK293T cells expressing α1β2γ2, α1(D219N)β2γ2, or

$\alpha1\beta2\gamma2$(R177G) $GABA_A$ receptors, assessed by bright field imaging. Representative images were shown from three biological replicates. (**D**) Effect of AA147 and AA263 treatment (5 μM, 24 h) on the mRNA level of $\alpha1$(D219N) subunits in HEK293T cells expressing $\alpha1$(D219N)$\beta2\gamma2$ $GABA_A$ receptors according to quantitative RT-PCR analysis (n = 3). DMSO (0.1% v/v final concentration) was used as a vehicle control. Each data point is reported as mean ± SEM. One-way ANOVA followed by post-hoc Tukey test was used for statistical analysis. *, $p < 0.05$; **, $p < 0.01$; ***, $p < 0.001$.

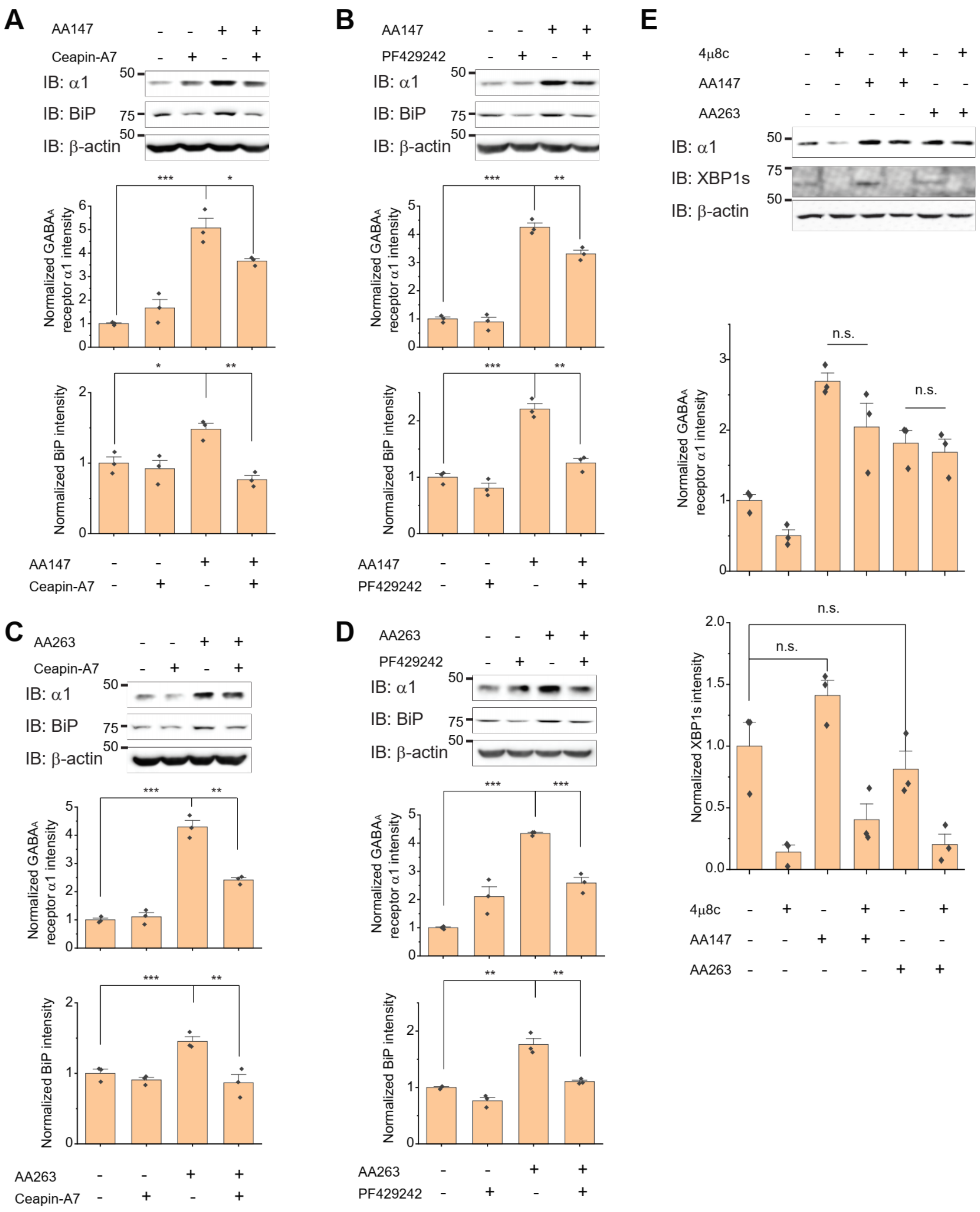

Supplementary Figure S4. **Contribution of the ATF6 pathway and the IRE1 pathway of the UPR for the effect of AA147 and AA263 on enhancing the α1(D219N) subunit protein levels.** (**A**) HEK293T cells stably expressing α1(D219N)β2γ2 $GABA_A$ receptors were treated with DMSO vehicle control, AA147 (5 μM, 24 h), Ceapin-A7 (10 μM, 24 h), or co-treated with

both AA147 (5 μM, 24 h) and Ceapin-A7 (10 μM, 24 h). (**B**) HEK293T cells stably expressing α1(D219N)β2γ2 $GABA_A$ receptors were treated with DMSO vehicle control, AA147 (5 μM, 24 h), PF429242 (10 μM, 24 h), or co-treated with both AA147 (5 μM, 24 h) and PF429242 (10 μM, 24 h). (**C**) HEK293T cells stably expressing α1(D219N)β2γ2 $GABA_A$ receptors were treated with DMSO vehicle control, AA263 (5 μM, 24 h), Ceapin-A7 (10 μM, 24 h), or co-treated with both AA263 (5 μM, 24 h) and Ceapin-A7 (10 μM, 24 h). (**D**) HEK293T cells stably expressing α1(D219N)β2γ2 $GABA_A$ receptors were treated with DMSO vehicle control, AA263 (5 μM, 24 h), PF429242 (10 μM, 24 h), or co-treated with both AA263 (5 μM, 24 h) and PF429242 (10 μM, 24 h). Ceapin-A7 and PF429242 are potent inhibitors of the ATF6 pathway. BiP is the protein marker for the activation of the ATF6 pathway. Quantification of the band intensities is shown on the bottom panels (n = 3). (**E**) HEK293T cells stably expressing α1(D219N)β2γ2 $GABA_A$ receptors were treated with DMSO vehicle control, AA147 (5 μM, 24 h), AA263 (5 μM, 24 h), 4μ8c (32 μM, 24 h), co-treated with AA147 (5 μM, 24 h) and 4μ8c (32 μM, 24 h), or co-treated with AA263 (5 μM, 24 h) and 4μ8c (32 μM, 24 h). 4μ8c is a potent inhibitor of the IRE1 pathway. XBP1s is the protein marker for the activation of the IRE1 pathway. Quantification of the band intensities is shown on the bottom panels (n = 3). Each data point is reported as mean ± SEM. One-way ANOVA followed by post-hoc Tukey test was used for statistical analysis. * $p < 0.05$; ** $p < 0.01$; *** $p < 0.001$; n.s. not significant.

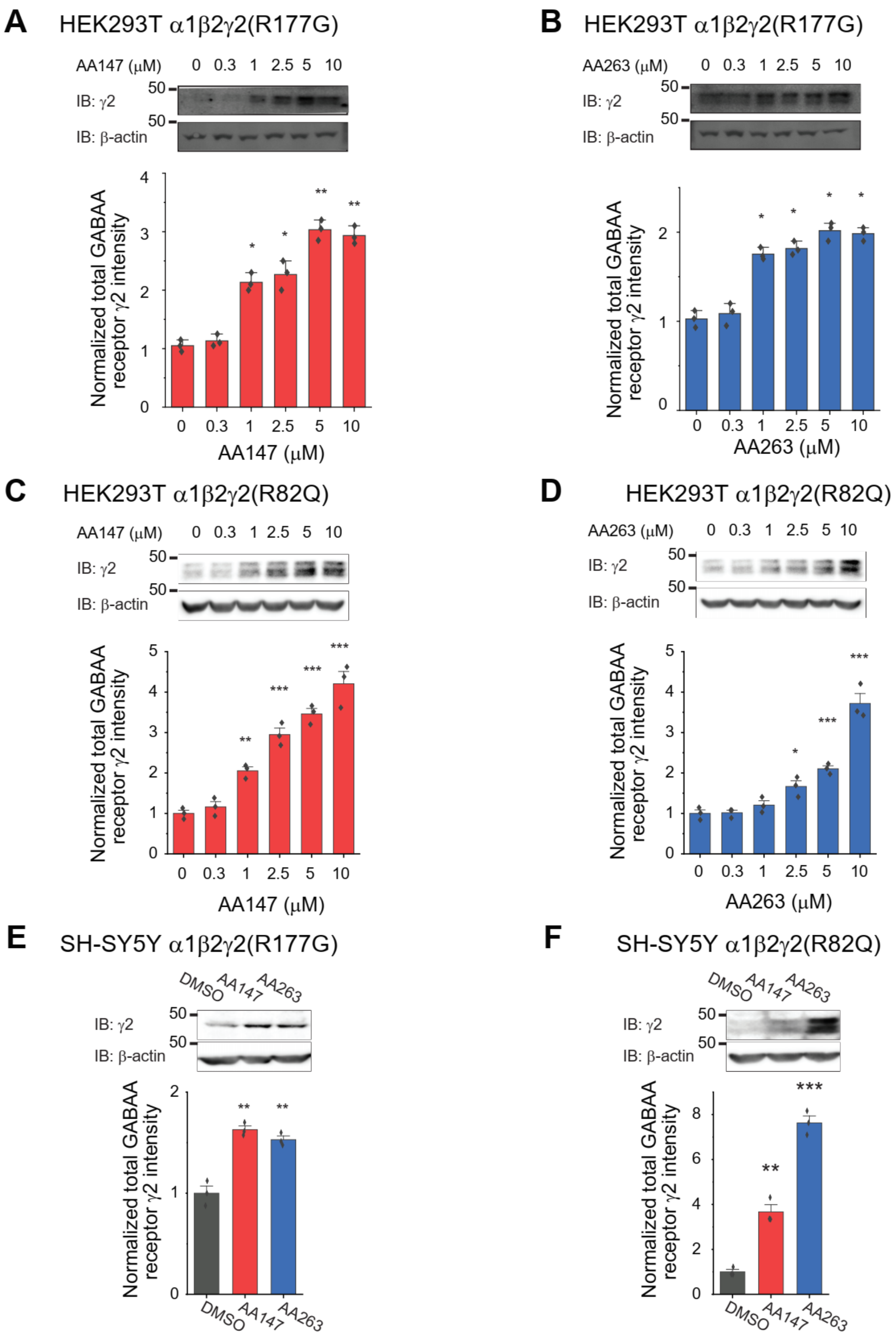

Supplementary Figure S5. Both AA147 and AA263 increase the total protein level of a variety of trafficking-deficient mutant receptors. Dose-response analysis of AA147 (**A**) and AA263 treatment (**B**) (24 h) in HEK293T cells expressing α1β2γ2(R177G). Dose-response

analysis of AA147 (**C**) and AA263 treatment (**D**) (24 h) in HEK293T cells expressing α1β2γ2(R82Q) $GABA_A$ receptors. Effect of AA147 (2.5 μM, 24 h) and AA263 (2.5 μM, 24 h) on the total protein level of γ2(R177G) subunits in SH-SY5Y cells stably expressing α1β2γ2(R177G) $GABA_A$ receptors (**E**), and γ2(R82Q) subunits in SH-SY5Y cells stably expressing α1β2γ2(R82Q) $GABA_A$ receptors (**F**). β-actin serves as total protein loading control. Quantification of the band intensities is shown on the bottom panels (n = 3). IB: immunoblotting. Each data point is reported as mean ± SEM. One-way ANOVA followed by post-hoc Tukey test was used for statistical analysis. *, $p < 0.05$; **, $p < 0.01$; ***, $p < 0.001$.

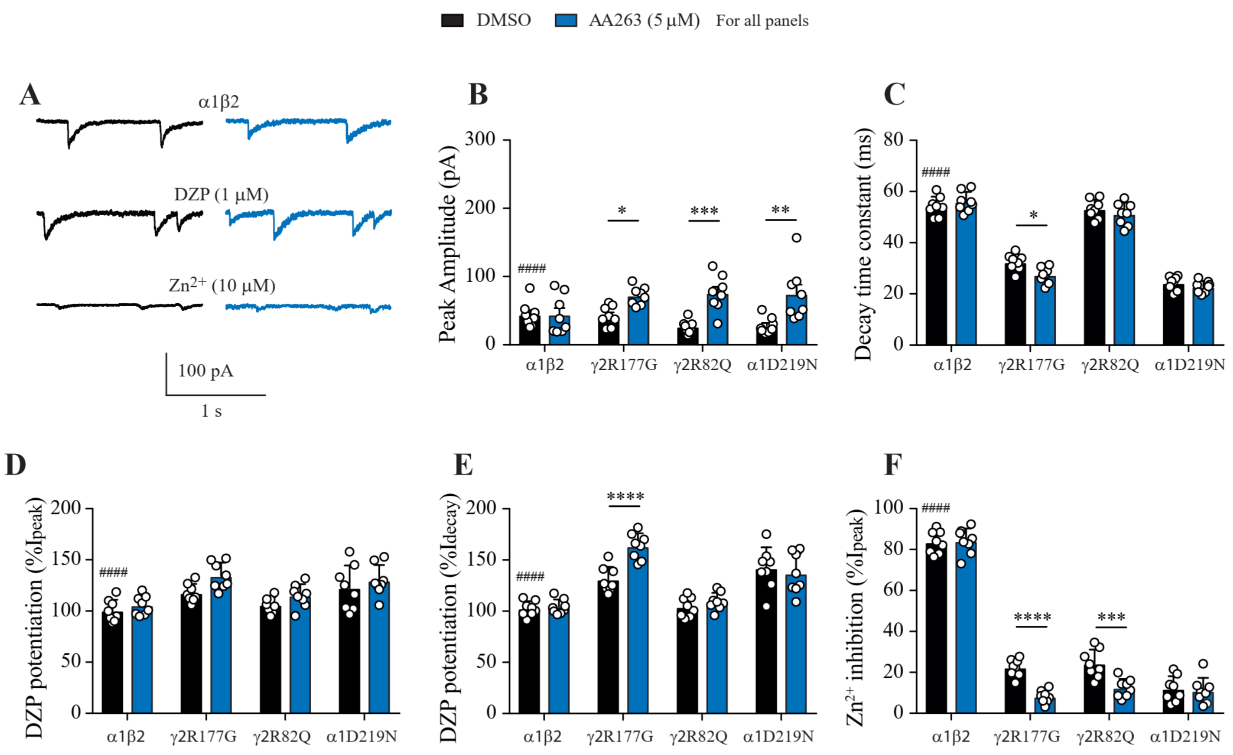

Supplementary Figure S6. IPSC propteries of α1β2 $GABA_A$ receptors. (**A**) Example IPSCs mediated by wild-type α1β2 $GABA_A$ receptors in control conditions (upper traces), in the presence of 1 μM DZP (middle traces) or 10 μM Zn^{2+} ions (lower traces). (**B-F**) Group bar plots showing the changes in peak amplitude (**B**), decay times (**C**), DZP potentiation of peak amplitude (**D**) or decay times (**E**) and inhibition by Zn^{2+} ions (**F**). For all panels, cells were treated with DMSO vehicle control (black traces) or AA263 (10 μM for 24 h, blue traces). The data for α1β2 $GABA_A$ receptors are compared to those for the triheteromeric receptors expressing the variants. Asterisks represent *p* values for the post-hoc comparisons of a two-way ANOVA with and without AA263 exposure, where * $p < 0.05$, ** $p < 0.01$, *** $p < 0.005$, **** $p < 0.001$. Number signs represent the *p* values for a one-way ANOVA without ATF6 activators exposure, where #### $p < 0.0001$. The ANOVA is compared to α1β2γ2 $GABA_A$ receptors.
